# Understanding patient-derived tumor organoid growth through an integrated imaging and mathematical modeling framework

**DOI:** 10.1371/journal.pcbi.1012256

**Published:** 2024-08-02

**Authors:** Einar Bjarki Gunnarsson, Seungil Kim, Brandon Choi, J. Karl Schmid, Karn Kaura, Heinz-Josef Lenz, Shannon M. Mumenthaler, Jasmine Foo

**Affiliations:** 1 Applied Mathematics Division, Science Institute, University of Iceland, Reykjavík, Iceland; 2 School of Mathematics, University of Minnesota, Twin Cities, Minnesota, United States of America; 3 Ellison Institute of Technology, Los Angeles, California, United States of America; 4 Keck School of Medicine, University of Southern California, Los Angeles, California, United States of America; 5 The Blake School, Minneapolis, Minnesota, United States of America; 6 Department of Biomedical Engineering, Viterbi School of Engineering, University of Southern California, Los Angeles, California, United States of America; University of California San Diego Division of Biological Sciences, UNITED STATES OF AMERICA

## Abstract

Patient-derived tumor organoids (PDTOs) are novel cellular models that maintain the genetic, phenotypic and structural features of patient tumor tissue and are useful for studying tumorigenesis and drug response. When integrated with advanced 3D imaging and analysis techniques, PDTOs can be used to establish physiologically relevant high-throughput and high-content drug screening platforms that support the development of patient-specific treatment strategies. However, in order to effectively leverage high-throughput PDTO observations for clinical predictions, it is critical to establish a quantitative understanding of the basic properties and variability of organoid growth dynamics. In this work, we introduced an innovative workflow for analyzing and understanding PDTO growth dynamics, by integrating a high-throughput imaging deep learning platform with mathematical modeling, incorporating flexible growth laws and variable dormancy times. We applied the workflow to colon cancer organoids and demonstrated that organoid growth is well-described by the Gompertz model of growth. Our analysis showed significant intrapatient heterogeneity in PDTO growth dynamics, with the initial exponential growth rate of an organoid following a lognormal distribution within each dataset. The level of intrapatient heterogeneity varied between patients, as did organoid growth rates and dormancy times of single seeded cells. Our work contributes to an emerging understanding of the basic growth characteristics of PDTOs, and it highlights the heterogeneity in organoid growth both within and between patients. These results pave the way for further modeling efforts aimed at predicting treatment response dynamics and drug resistance timing.

## Author summary

Patient-derived tumor organoids (PDTOs) are miniaturized models of tumors, developed using a patient’s own tumor tissue, which can be grown outside of the body in a laboratory setting. PDTOs enable researchers to better understand tumor biology and to model how an individual’s tumor may respond to various cancer treatments. In this work, by integrating PDTOs with dynamic imaging and mathematical modeling, we develop a method for investigating the fundamental laws of tumor organoid growth on a patient-by-patient basis (S1 Fig). We identify a simple mathematical model which applies to the growth of PDTOs derived from three different patients, and we quantify variability in organoid growth both within and between patients. Our work is ultimately motivated by the potential of combining mathematical modeling with drug screening data for personalized treatment optimization.

## Introduction

Patient-derived tumor organoids (PDTOs) are a valuable cell culture model system for studying dynamic tumor cell growth, tissue-specific cellular differentiation and cell-cell interactions. PDTOs mimic features of the *in vivo* microenvironmental conditions and support physiologically relevant drug testing [1–4]. PDTOs also maintain the genetic and phenotypic features of patient tumor tissues, overcoming many limitations of traditional preclinical models by recapitulating both intra- and interpatient heterogeneities. Target identification and selection of effective treatments using PDTOs can pave the way for functional precision medicine approaches based on genetic and environmental factors [5, 6]. Indeed, the recent FDA modernization Act 2.0 expedites the use of alternative models to replace, reduce and refine current animal model testing in pre-clinical drug studies [7]. 3D cell culture models may reduce the costs associated with drug discovery and help mitigate the poor clinical translation of laboratory results in oncology [8].

PDTOs can be combined with multiple assay methods and analysis tools to enable high-throughput and high-content investigations of patient-specific heterogeneities and tumor microenvironmental interactions. Advances in 3D imaging techniques have been used to examine quantitative phenotypic changes in organoid models subjected to environmental and drug perturbations [9, 10]. Additionally, the surge in machine learning and deep learning techniques for image analysis presents novel approaches to analyze extensive 3D imaging data [11–14]. However, standard solutions for measuring and interpreting spatial and temporal dynamics of PDTOs are limited. Quantification of these dynamics will provide additional insights into the complex processes governing tissue development, disease progression, and response to treatment.

For decades, mathematical modeling has proven to be useful for understanding cancer initiation, tumor progression and the evolution of drug resistance [15–21], as well as for developing new clinical strategies [22–25]. In the context of precision medicine, mathematical and computational modeling can aid in the drug discovery process [26, 27] and in the selection of personalized treatment strategies [28–30]. A crucial first step toward this goal is to understand the basic mathematical properties of tumor growth in the untreated condition. A large stream of literature has applied classical growth models like the exponential, power law, Gompertz, logistic and von Bertalanffy models to tumor data from human patients, animal models and in vitro tumor spheroids. These investigations, which are reviewed in Section 1 of S1 Text, indicate that the most appropriate growth model is context-dependent. For patient-derived organoids, the recent review by Montes-Olivas and colleagues [31] points out that relative to advancements in the development of experimental protocols, mathematical and computational models of organoid growth remain comparatively underdeveloped. These models are usually agent-based computational models which describe the spatial dynamics of stem cell differentiation within the organoids, often taking into account the signaling dynamics of key cell fate regulators [31]. For the specific case of colon cancer organoids, Yan et al. [32] recently developed a mathematical model involving stem, committed progenitor and terminally differentiated cells. Their results show that the dynamics of organoid growth are highly dependent on quantitative parameters such as the mitosis rate of stem cells and the strength of positive and negative feedback loops, as well as the presence of external signaling factors. More recently, Montes-Olivas et al. [33] modified an existing two-dimensional agent-based model [34] to simulate the formation of budding structures in intestinal organoids.

The main goal of this work is to combine quantitative imaging with mathematical modeling to develop a novel integrated pipeline for studying the growth characteristics of PDTOs. Our pipeline incorporates label-free, deep learning image analysis that provides multi-parametric information of tracked individual PDTOs over time. We apply the integrated imaging–modeling method to experimental data involving colon cancer organoids derived from three different patients, with the aim of characterizing the fundamental growth characteristics of organoids using classical mathematical growth models. Throughout, we place special emphasis on analyzing the heterogeneity in organoid growth both within and between patients. Our work is ultimately motivated by the potential of combining mathematical modeling with high-throughput drug screening data for drug discovery, drug testing and personalized treatment optimization. Therefore we aim to identify simple models of untreated organoid growth that are appropriate for the level of data resolution attainable in the high-throughput setting.

## Materials and methods

### Ethics statement

The use of patient samples was reviewed and approved by the USC Biomedical Institutional Review Board Committee, under protocol number HS-06–00678. All patients provided written consent prior to sample collection. Samples were de-identified to research staff.

### Patient tissue processing and organoid cultures

We developed organoids using three different CRC patient samples in this study. Patient information was de-identified and randomized internal IDs (000UP, 000US and 000UK) were assigned to each patient sample. To simplify, the first 3 digit number, 000, was omitted in our study. The genomic analyses for each patient sample are described in Table 1. The CRC patient tissues (UP, US, UK) were processed as described previously in [35]. Briefly, tumor tissue was enzymatically digested into single cells and dissociated cells were seeded into 3D extracellular matrix (ECM) gel (Cultrex Reduced Growth Factor Basement membrane extract, Type 2, BME) with media tailored to form and maintain tumor organoids. H2B-GFP labeled US organoids were generated by lentiviral transduction [35] and used to create an organoid image dataset labeled with ground-truth live and dead classifications for neural network (NN) training. Organoid cultures were maintained in 24-well plates and passaged by mechanical breakdown with pipetting in the Gentle cell dissociation reagent (StemCell). For single cell dissociation, TrypLE (ThermoFisher) digesting solution (1:1, TrypLE:PBS with 1:1,000 Y-27632 (StemCell)) was used. TruSight Oncology 500 assay (Illumina) and Tempus xT V4 pannel (Tempus) were used to detect DNA mutations in cancer-related genes.

**Table 1:** Patient-derived sample information.

PDO Name	UK	UP	US
Tumor Stage	pT3N2bM1a (Stage 4A)	pT4bN1aM1a (Stage 4)	pT4aN0Mx (Stage 2)
Tissue Type	Colon	Colon	Colon
Mutations from Tissue Sequencing	PI3KCA, **TP53**, AR, **ALK**, **EPHA5**, MDC1, PTPRS, CD3EAP, PTPRT, NCOA3, FANCA, PARP1, MED12, PREX2	**TP53**, **ASXL1**, **RUNX1**, **FOXP1**, **SOCS1**, **CIC**, **CCND3**, **DNMT3B**, **PTPRT**, PAX7, ARID5B, VTCN1, RFWD2	**KRAS**, **TP53**, **SMAD4**, **ERBB4**, MAP3K1, AXL, PBRM1, SH2B3, CIC, **MCL1**, AMER1, **PTPRD**, **GATA6**, NCOR1, INPP4A
CNVs (Tissue)	FGFR1 (2.072), MYC (2.588)	BRCA2 (1.437), ERBB2 (1.516), FGF14 (1.659)	BRCA2 (1.539), FGF14 (1.538), FGFR4 (1.595), PDGFRB (1.419)
MSI (Tissue)	3.33	0.87	0.88
TMB (Tissue)	1.6	4.7	7.1
Organoid Morphology	Cryptic	Cryptic	Cryptic
Mutations from Organoid Sequencing	APC,NF1, **TP53**, **ALK**, **EPHA5**, DNMT1, MCL1, CTLA4, ANKRD26, CD3EAP	**TP53**, APC, **ASXL1**, **RUNX1**, **FOXP1**, SH2B3, **SOCS1**, **CIC**, **CCND3**, MDM2, **DNMT3B**, **PTPRT**, **MCL1**	APC, **KRAS**, **TP53**, **SMAD4**, FLT3, **ERBB4**, EP300, SH2B3, ZFHX3, KAT6A, **MCL1**, **PTPRD**, **GATA6**
CNVs (Organoid)	AKT2 (1.604), BRCA2 (1.576), CCNE1 (1.477), EGFR (2.203), ERBB2 (1.45), ERCC2 (1.788), JAK2 (1.542)	BRCA2 (1.513), CDK4 (1.548), EGFR (1.73), ERBB3 (1.461), FGF10 (1.671), FGF23 (1.55), FGF6 (1.556), KRAS (1.464), LAMP1 (1.454), MET (1.62), RICTOR (1.787)	BRCA2 (1.872), CDK4 (1.515), FGF14 (1.666), FGF9(1.651), FGFR4(1.739), LAMP1 (1.645), PDGFRB (1.586)
MSI (Organoid)	1.67	3.33	0.84
TMB (Organoid)	1.6	9.4	7.1

Tumor stage and molecular alterations from sequenced tissues and organoid samples are listed including, single nucleotide variants (SNVs), insertions and deletions (InDels), copy number variations (CNVs), micro-satellite instability (MSI) score and tumor mutational burden (TMB). CNVs larger than 1.3 are considered as significant. MSI is shown as percentages of unstable sites, with over 20 percent considered as MSI high. TMB score is shown as a number of mutations per megabase of DNA. Over 10 mutations per megabase of DNA is considered as TMB high. Common mutated genes between tissue and organoid were highlighted with bolded texts.

Two UK datasets were produced, with experiments conducted on 12.09.2022 (UK–1) and 12.16.2022 (UK–2). Two UP datasets were produced, with experiments conducted on 11.04.2022 (UP–1) and 12.09.2022 (UP–2). Three US datasets were produced, with experiments conducted on 08.26.2022 (US–1) and 11.04.2022. On the latter date, two experiments were performed which were conducted by different researchers (US–2 and US–3). These datasets are collectively referred to as the UK/UP/US datasets. Additional data from a previously published dataset, the US-GFP organoid dataset (Plate 1 and Plate 2) [35] was also used in our analyses.

### Confocal 3D live cell imaging and quantitative image analysis

1,000 dissociated single cells of each organoid line were seeded into each well of a 96 multi-well plate with BME. After 4 days of culturing, confocal imaging (Evident-Olympus FV3000 microscope) was performed with multiple Z scans to capture the entire organoid culture area on three different timepoints (Days 0, 3, 5). cellSens (Evident-Olympus) software was used to create 2D projection images using the extended focal imaging (EFI) process and perform live-dead organoid classification with the pre-trained neural network (NN). The labeled NN training image dataset was prepared with H2B-GFP-labelled US organoids with and without 1 μM staurosporine (ST) treatment. To generate the training dataset, each organoid was segmented from the GFP channel, and live and dead organoids were classified in untreated control and ST-treated groups, respectively. The brightfield (Transmitted) channel was used as an input to train the NN (Standard U-net). The training was done with 75,000 iterations and an optimal checkpoint was selected and saved after confirming with validation images.

Brightfield organoid image datasets were collected from UP, US and UK organoids. Three timepoint (Days 0, 3, 5) images were combined as a time series and EFI processing was performed to generate 2D projected images. Automatic NN batch processing segmented label-free brightfield images of organoids while simultaneously classifying each detected object as live or dead. Each organoid was tracked over time to examine longitudinal organoid growth and morphological changes. Organoid size (area), sphericity, shape factor, convenity, XY position and tracking information were exported as spreadsheets and used to test different growth models. R 4.3.2 and GraphPad Prism 10 was used to generate graphs and analysis.

For S2 Fig and S1 Video, live cell imaging of US-GFP organoids was performed with Operetta high-content imaging system (PerkinElmer) using 20x objective. Single Z plane (middle) images were taken every 20 minutes for 12 hours. Additionally for immunostaining, organoid cultures were fixed with 4 percent of paraformaldehyde (PFA) and stained with anti-rabbit Ki67 (1:500, Abcam), anti-mouse E-cadherin (1:300, Cell signaling) and DAPI (1:60,000). Alexa-488 and Alexa-555 conjugated secondary antibodies were used. Confocal 3D imaging was performed with the Evident-Olympus FV3000 system.

### Conversion from organoid area to cell number

As the mathematical models we employ are intended to model the number of cells or individuals in a population, we convert the area measurements produced by the NN into cell number estimates before fitting the mathematical models to the UK/UP/US datasets. First, we convert organoid area to organoid volume. For the conversion, we assume that each organoid is an ellipsoid, which implies that its two-dimensional projection is an ellipse with axes *a* and *b* and area *A* = *πab*. We furthermore assume that the third axis, which is not ascertainable from the 2D projected images, is the geometric mean of the other two axes, $c=\sqrt{ab}$. Under these assumptions, the volume of an organoid can be written in terms of its area as

$$\begin{array}{c} V=\frac{4}{3}\pi abc=\frac{4}{3\sqrt{\pi }}A^{3/2}. \end{array}$$

Alternative volume conversion methods are discussed in Section 4 of S1 Text. We then convert the volume estimate for each organoid to an estimate of the number of live cells in the organoid. For the conversion, we refer to the US–GFP dataset, which includes data both on the volume and the number of live cells in each organoid. We computed the ratio between the volume and the number of live cells for each organoid on Day 0, which has a median value of 7,208 *μ*m^3^. We did the same calculation on Days 1, 3 and 6 and observed similar median values. Then, for each organoid in the UK/UP/US datasets, we divide the volume estimate obtained as above by 7,208 *μ*m^3^ to get an estimate of the number of live cells in the organoid.

### Mathematical models

**Fig 1:**
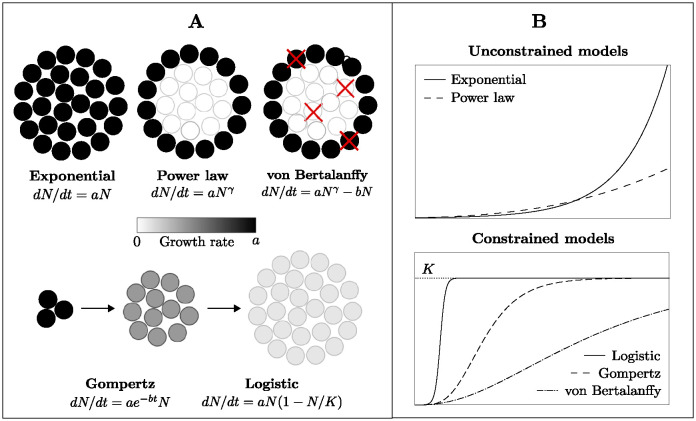
Overview of mathematical models. A: The exponential model assumes that all cells in the organoid divide at the same rate *a*, while the power law model assumes that cell divisions are restricted to a subset of cells, for example at the surface of the organoid. The Gompertz and logistic models each assume that initial growth is exponential at rate *a* and that the growth slows down over time. This can either be due to the cell division rate decreasing uniformly across the organoid, or due to a decreasing subset of actively dividing cells over time. The von Bertalanffy model assumes that cell proliferation follows the power law model, but in addition that cells die uniformly across the organoid at rate *b*. B: The exponential and power law models are models of unconstrained growth, while the Gompertz, logistic and von Bertalanffy models all assume that the growth eventually stops, with the organoid reaching a so-called “carrying capacity” *K*.

Each mathematical model we employ is defined by a differential equation for *N*(*t*), the number of live cells in the organoid at time *t*. In the first five subsections below, we describe the models, and in the final subsection, we state a general differential equation from which all the models can be derived. A visual overview is given in Fig 1. The exponential and power law models are unconstrained growth models, in the sense that the population grows without bound as time passes (*N*(*t*) → ∞ as *t* → ∞). The Gompertz, logistic and von Bertalanffy models are “S-shaped” growth models, where the population size eventually converges to a so-called “carrying capacity”. We refer to Section 1 of S1 Text for a review of previous works applying these mathematical models to data from human patients, animal tumor models and tumor spheroids.

#### Exponential model

The first model we consider is the exponential growth model, which assumes that each cell in the organoid divides at the same rate *a* > 0. Equivalently, it can be assumed that a constant proportion of cells actively divides at any time. Under the exponential model, the time evolution of organoid cell number is described by the differential equation

$$\begin{array}{cc} & \frac{dN}{dt}=aN,\;N\left(0\right)=N_{0}, \end{array}$$ (1)

which has the explicit solution

$$\begin{array}{cc} & N\left(t\right)=N_{0}e^{at},\;t\geq 0. \end{array}$$ (2)

We note that the differential equation Eq 1 can also be viewed as describing the average dynamics of a stochastic growth model where cell divisions are asynchronous and cell cycle lengths are assumed to be independent exponential random variables with rate *a* > 0.

#### Power law model

The second model we consider is the power law growth model. As opposed to assuming that all cells in the organoid divide at the same rate, the power law model assumes that only a subset of cells, which has a lower spatial dimension than the full organoid, is actively dividing at any time. This can for example happen when cell divisions are restricted to the surface of the organoid. The power law model is described by the differential equation

$$\begin{array}{cc} & \frac{dN}{dt}=aN^{\gamma },\;N\left(0\right)=N_{0}, \end{array}$$ (3)

with 0 < *γ* < 1, which has the explicit solution

$$\begin{array}{cc} & N\left(t\right)={\left(N_{0}^{1-\gamma }+\left(1-\gamma \right)at\right)}^{1/(1-\gamma )},\;t\geq 0. \end{array}$$ (4)

Note that the power law differential equation Eq 3 can be viewed as a generalization of the exponential differential equation Eq 1. If the organoid grows as a three-dimensional ball, then taking *γ* = 2/3 produces a model where cell divisions only occur at its (two-dimensional) surface. In our analysis, we consider three power law models: *γ* = 1/2, *γ* = 2/3 and *γ* = 3/4, as we further explain in Section “von Bertalanffy model”.

#### Gompertz model

The third model we consider is the Gompertz model, where it is assumed that an initial exponential growth rate *a* > 0 decays exponentially over time according to a decay parameter *b* ≥ 0. In other words, the time evolution of the model is given by the differential equation

$$\begin{array}{cc} & \frac{dN}{dt}=ae^{-bt}N,\;N\left(0\right)=N_{0}, \end{array}$$ (5)

which has the explicit solution

$$\begin{array}{cc} & N\left(t\right)=N_{0}\;\text{exp}(\left(a/b\right)\left(1-e^{-bt}\right)),\;t\geq 0. \end{array}$$ (6)

As *t* → ∞, the Gompertz model reaches a carrying capacity *K* ≔ *N*_0_*e*^*a*/*b*^. An alternative formulation of the Gompertz model, which involves an initial growth rate *α* and the carrying capacity *K*, is given by the differential equation

$$\begin{array}{cc} & \frac{dN}{dt}=\alpha \;\text{log}\left(K/N\right)N=\alpha \;\text{log}\left(K\right)N-\alpha N\;\text{log}\left(N\right),\;N\left(0\right)=N_{0}, \end{array}$$ (7)

which has the explicit solution

$$\begin{array}{cc} & N\left(t\right)=K\;\text{exp}(\;\text{log}\left(N_{0}/K\right)e^{-\alpha t}),\;t\geq 0. \end{array}$$ (8)

#### Logistic model

The fourth model we consider is the logistic growth model. This model also involves an initial exponential growth rate *a* > 0, but here, the growth rate decays linearly with the size of the population until the population reaches a carrying capacity *K*. In other words, the logistic model is given by the differential equation

$$\begin{array}{cc} & \frac{dN}{dt}=aN(1-N/K)=aN-aN^{2}/K,\;N\left(0\right)=N_{0}, \end{array}$$ (9)

which has the explicit solution

$$\begin{array}{c} N\left(t\right)=N_{0}K{\left(N_{0}+\left(K-N_{0}\right)e^{-at}\right)}^{-1},\;t\geq 0. \end{array}$$ (10)

We note that the logistic differential equation can be viewed as a nonspatial single-species competition model, where the frequency of interactions between cells is on the order of *N*^2^, and population growth is impeded proportionally to the frequency of interactions.

In both the Gompertz and logistic models, the division rate of cells in the organoid is assumed to decrease over time. Alternatively, it can be assumed that a smaller and smaller proportion of cells actively divides as the organoid grows. This can for example be due to lack of nutrients or growth factors, spatial limitations or an increasing level of cell interference. The main difference between the two models is that the logistic model is symmetric, where the initial and final growth phases mirror one another, while the Gompertz model is asymmetric.

#### von Bertalanffy model

The final model we consider is the von Bertalanffy growth model. In the classical version of the model, the organoid is assumed to grow as a three-dimensional ball and cell divisions are assumed to occur only at the surface of the organoid, with cell deaths occurring uniformly across the organoid at some rate *b* > 0. In other words, the time-evolution of the model is given by the differential equation

$$\begin{array}{c} \frac{dN}{dt}=aN^{2/3}-bN,\;N\left(0\right)=N_{0}. \end{array}$$

A broader version of the von Bertalanffy model assumes more generally that the subset of actively dividing cells has a lower spatial dimension than the full organoid, i.e. that cell proliferation follows a power law (Section “Power law model”). This leads to the differential equation

$$\begin{array}{cc} & \frac{dN}{dt}=aN^{\gamma }-bN,\;N\left(0\right)=N_{0}, \end{array}$$ (11)

for 0 < *γ* < 1, which has the explicit solution

$$\begin{array}{cc} & N\left(t\right)={\left(\left(a/b\right)+\left(N_{0}^{1-\gamma }-a/b\right)e^{-(1-\gamma )bt}\right)}^{1/(1-\gamma )},\;t\geq 0. \end{array}$$ (12)

As *t* → ∞, the generalized von Bertalanffy model reaches a carrying capacity *K* ≔ (*a*/*b*)^1/(1 − *γ*)^. Reparametrizing in terms of *b*, *γ* and *K*, Eq 12 can be rewritten as

$$\begin{array}{cc} & N\left(t\right)=K{\left(1+\left({\left(N_{0}/K\right)}^{1-\gamma }-1\right)e^{-(1-\gamma )bt}\right)}^{1/(1-\gamma )},\;t\geq 0. \end{array}$$ (13)

Same as for the power law models, in our analysis, we consider three versions of the von Bertalanffy model, with *γ* = 1/2, *γ* = 2/3 and *γ* = 3/4. The latter two choices are biologically motivated, with *γ* = 2/3 corresponding to the classical von Bertalanffy model and *γ* = 3/4 corresponding to the general ontogenetic growth model of West et al. [36]. This is further discussed in our mathematical modeling review, Section 1 of S1 Text.

#### Relationship between the models

We note that all of the growth models discussed in the previous subsections can be derived from a differential equation of the form

$$\begin{array}{c} \frac{dN}{dt}=aN^{\gamma }-bN^{\delta }, \end{array}$$ (14)

where *a* > 0, *b* ≥ 0 and *γ* < *δ*. For the exponential model, *b* = 0 and *γ* = 1, and for the power law model, *b* = 0 and 0 < *γ* < 1. For the logistic model, *b* = *a*/*K*, *γ* = 1 and *δ* = 2, and for the von Bertalanffy model, 0 < *γ* < 1 and *δ* = 1. The Gompertz model cannot be written directly in the form of Eq 14, but when properly reparametrized, it emerges as a limiting case of Eq 14 as *γ* → *δ* [37, 38]. In particular, if we rewrite Eq 14 as

$$\begin{array}{c} \frac{dN}{dt}=cN^{\delta }-dN^{\delta -\varepsilon }\frac{N^{\varepsilon }-1}{\varepsilon }, \end{array}$$ (15)

where *ε* = *δ* − *γ* > 0, *d* = *a*(*δ*− *γ*) > 0 and *c* = *a* − *b*, and send *ε* → 0, we get

$$\begin{array}{c} \frac{dN}{dt}=cN^{\delta }-dN^{\delta }\;\text{log}\left(N\right). \end{array}$$ (16)

This differential equation has the form of the Gompertz differential equation Eq 7 if we take *δ* = 1. Thus, the von Bertalanffy model with *γ* = 1 − *ε* where *ε* > 0 is small and *a* > *b* approximates the Gompertz model with the appropriate reparametrization of *a* and *b*. This is one reason we do not consider the von Bertalanffy model with *γ* above 3/4, as is further discussed in Section 3 of S1 Text.

### Variable dormancy time

In the UK/UP/US datasets, organoids are seeded as single cells on Day −4, and cell number estimates based on two-dimensional area measurements are obtained for each organoid on Days 0, 3 and 5 (Section “Conversion from organoid area to cell number”). In the US–GFP dataset, organoids are seeded as single cells on Day −7, and the number of live cells in each organoid is available on Days 0, 1, 3 and 6. When fitting the mathematical models to the data (Section “Mathematical models”), we assume that each organoid starts growing from a single cell at some time before Day 0. As there is significant heterogeneity in organoid size on Day 0, both within and between patients, we allow the time at which the organoid starts growing to be variable. This modeling decision also reflects the fact that after single cells are extracted from the patient tissue and seeded into the 3D extracellular matrix with growth media, it can take them varying amounts of time to adjust to the new environment. More precisely, we assume that each organoid starts growing from a single cell on Day −*τ*, where *τ* is an organoid-specific parameter. For the UK/UP/US datasets, we allow *τ* to be any number in the range 0 ≤ *τ* ≤ 4, and for the US–GFP dataset, we allow 0 ≤ *τ* ≤ 7. Since each model is started with a single cell, *N*_0_ = 1.

### Data filtering

There is still no clear definition concerning the specific size and cell number at which 3D cell aggregates are considered organoids. Before fitting the mathematical models to the UK/UP/US datasets (Section “Mathematical models”), we removed any tracked object identified as being dead on Day 3 or Day 5, and any tracked object with an area below 300 *μ*m^2^ on Days 0, 3 or 5, to remove any smaller size cell clusters and debris. We furthermore only consider organoids increasing in estimated cell number between each pair of time points, since our aim is to study growing organoids, and our mathematical models are intended for that purpose. We finally apply one additional level of filtering intended to alleviate segmentation errors made by the NN image analysis. These errors include the misidentification of two organoids that overlap in the 2D projected images as a single organoid, and the misidentification of a single large and differentiated cryptic organoid as two or more smaller organoids. See further Section 2 of S1 Text. The results of each filtering step and the number of organoids included in the final analysis are shown in S1 Table.

### Model fitting

For each individual organoid and each growth model, the model parameters ***θ*** and the starting time parameter *τ* are estimated by minimizing the sum of squared errors between the model prediction and the data. More precisely, if *n*_1_, …, *n*_*k*_ are cell number estimates collected on Days *t*_1_, …, *t*_*k*_, and *N*(*t*; ***θ***, *τ*) is the model prediction at time *t*, the parameters are estimated as

$$\begin{array}{c} \left(\hat{\theta },\hat{\tau }\right)≔{\text{argmin}}_{\theta ,\tau }\varphi^{2}(\theta ,\tau ), \end{array}$$ (17)

where

$$\begin{array}{c} \varphi^{2}(\theta ,\tau )≔\sum_{i=1}^{k}(n_{i}-N(\tau +t_{i};\theta ,\tau ){)}^{2}. \end{array}$$

Note that time 0 in the model is Day −*τ* in the experiments. Also note that *N*(0) = *N*_0_ = 1. The estimate Eq 17 is the maximum likelihood estimate for the statistical model

$$\begin{array}{c} n_{i}=N\left(\tau +t_{i};\theta ,\tau \right)+\varepsilon_{i}, \end{array}$$ (18)

where *ε*_1_, …, *ε*_*k*_ are independent and identically distributed *N*(0, *σ*^2^) random variables with *σ*^2^ > 0. To see why, note that the log-likelihood function for Eq 18 is

$$\begin{array}{c} \text{log}\;L(\theta ,\tau ,\sigma )=-\frac{k}{2}\;\text{log}\left(2\pi \sigma^{2}\right)-\frac{1}{2\sigma^{2}}\varphi^{2}\left(\theta ,\tau \right). \end{array}$$ (19)

This function is maximized with respect to (***θ***, *τ*) by $\left(\hat{\theta },\hat{\tau }\right)$ as defined in Eq 17. The optimal value for *σ*^2^ > 0 can be computed as ${\hat{\sigma }}^{2}≔(1/k)\varphi^{2}(\hat{\theta },\hat{\tau })$.

For each organoid and each growth model, the minimization in Eq 17 is performed 1,000 times using *fmincon* in MATLAB, starting from different random guesses for the parameters in question. For all supplementary analyses in S1 Text, the minimization is performed 500 times.

The statistical model Eq 18 assumes that the Gaussian errors *ε*_1_, …, *ε*_*k*_ are additive and that they have the same magnitude at each time point. However, since the organoids being modeled are growing over time, it is also reasonable to assume that the magnitude of the error scales with organoid size. In Section 5 of S1 Text, we ensure that our main results continue to hold under a logarithmic transformation of the models and data, which leads to a multiplicative error that scales with organoid size.

### Model selection

If we include the starting time parameter *τ*, the exponential and power law models (the unconstrained models) have two parameters, while the Gompertz, logistic and von Bertalanffy models (the constrained models) have three parameters. To account for differences in model complexity, we use the Bayesian information criterion (BIC) to evaluate model fit quality. For the statistical model Eq 18, the BIC is given by

$$\begin{array}{cc} \text{BIC} & =-2\;\text{log}\;L\left(\hat{\theta },\hat{\tau },\hat{\sigma }\right)+\left(p+1\right)\;\text{log}\left(k\right)\\ & =k\left(1+\;\text{log}\left(2\pi \right)\right)+k\;\text{log}(\varphi^{2}\left(\hat{\theta },\hat{\tau }\right)/k)+\left(p+1\right)\;\text{log}\left(k\right), \end{array}$$

where *p* is the number of parameters in the model. Due to the limited number of datapoints per organoid, the error $\varphi^{2}\left(\hat{\theta },\hat{\tau }\right)$ is small for many organoids. In our analysis, we set 10^−6^ as the smallest possible value for the error and consider it effectively zero error.

When evaluating model fit quality, we also use the mean normalized fitting error across all organoids in each dataset. The normalized fitting error is a simpler metric which is computed for each individual organoid as

$$\begin{array}{c} {\left(\frac{1}{k}\sum_{i=1}^{k}n_{i}\right)}^{-1}\sqrt{\sum_{i=1}^{n}{\left(n_{i}-N\left(\tau +t_{i};\theta ,\tau \right))\right)}^{2}}. \end{array}$$

The normalization accounts for the fact that the sizes of individual organoids vary across orders of magnitude. It also gives an easily interpretable error measurement, since the mean normalized error can be viewed as the mean percentage error in the estimation.

## Results

### Patient-specific organoid size and morphological changes can be measured by AI-driven image analysis with individual organoid tracking

We used three different colorectal cancer (CRC) patient organoids (UK, UP, and US) with distinct clinical and genomics signatures as described in Table 1 (Patient-derived sample information). Gene mutations, copy number variation, tumor mutational burden, and microsatellite instability status were determined for each PDTO using next generation sequencing. We observed agreement in gene mutations between each patient organoid and the corresponding tumor tissue, indicating that PDTOs serve as a suitable model for the patient’s tumor tissue. Taken together, inter-patient heterogeneity, reflective of the CRC clinical population, is apparent in the PDTOs selected for this study.

**Fig 2:**
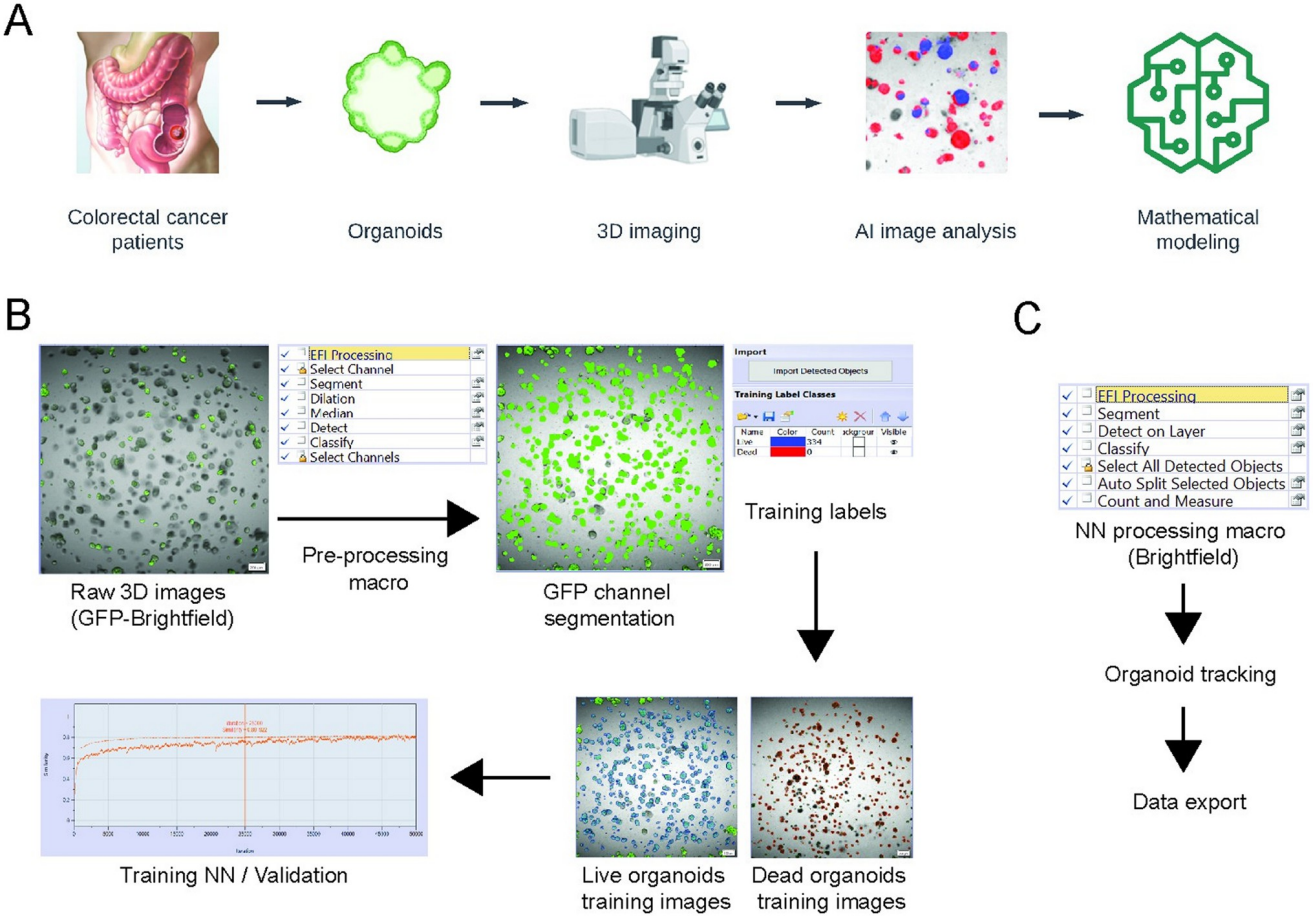
Overview of organoid mathematical modeling. A: Colorectal cancer patient organoids were imaged at multi-time points and analyzed using an AI-based method. The exported results were analyzed using our mathematical models. The diagram was created using LucidChart and BioRender software. B: NN training with H2B-GFP US organoid data. Pre-processing with extended focal imaging was done with an automated macro. Live/dead organoid training labels were generated from untreated and 1uM ST-treated organoids. Training was performed with 750,000 iterations and validated with auto-selected images. C: NN processing with brightfield images and tracking. NN processing and data export were automated with macro for batch processing.

To analyze organoid growth and morphology features, we established a novel pipeline combining high-throughput PDTO experiments with deep learning-based image analysis and mathematical modeling of organoid growth dynamics. The overall workflow, involving the processes of organoid development, 3D imaging and image analysis, and mathematical modeling is visualized in Fig 2A. To generate our deep-learning neural network (NN) training dataset, we utilized H2B-GFP labeled US PDTOs that were generated by lentiviral transduction in our previous study [35], since the GFP signal improves organoid detection and segmentation. Specifically, an extended focal imaging (EFI) method was used to project multi-z stack images to generate 2D images. After pre-processing, individual organoids were labeled as either live (Untreated) or dead (1 uM Staurosporine-treated). These labels were used to train a NN based on a standard U-Net (Fig 2B).

The resulting trained NN was then applied to new label-free PDTO images. We prioritized label-free imaging to optimize the speed and throughput of data collection. Specifically, new brightfield channel images at different timepoints were treated as a time series (Day 0, 3, 5) and segmented using the trained NN with an automated macro process. Individual organoid tracking was performed with the NN segmented layer to follow organoid changes over time. Organoid size (area) and morphology (shape factor, sphericity, convexity) measurements were exported to generate graphs for visualization (Fig 2C). This multi-timepoint analysis enabled exploration of the temporal dynamics of organoid growth using mathematical modeling, the results of which are discussed in the following sections.

**Fig 3:**
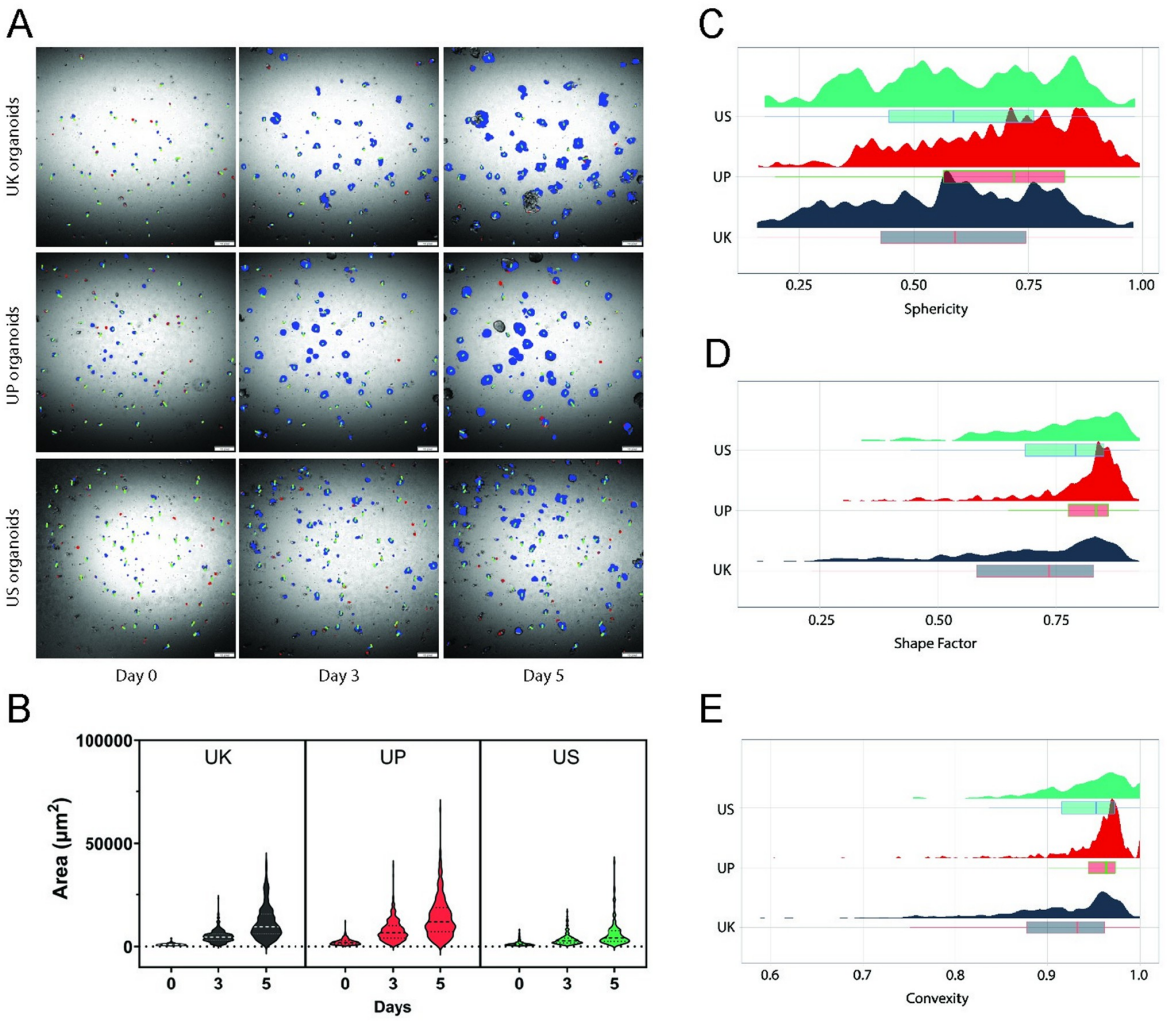
Organoid tracking and morphology measurement. A: Each patient organoid segmented with NN. Representative images show the changes of tracked organoids over time. Blue: live organoids, Red: dead organoids, Green: tracks. B: Area measurements of tracked individual UK, UP and US organoids at Day 0, 3 and 5. Thick dotted lines = Median, Thin dotted lines = Quartile. C: Distribution of sphericity at Day 5. Sphericity is approximately the squared quotient of width and length. D: Distribution of shape factor at Day 5. The shape factor is an area relative to the area of a circle with an equal perimeter. E: Distribution of convexity at Day 5. The convexity is an area relative to the area of object’s convex hull. UK = 303 organoids, UP = 496 organoids, US = 136 organoids.

Fig 3A highlights the inter-patient differences in area and morphological changes over time for each patient organoid (S2, S3 and S4 Videos). UK and UP organoids are on average larger than US organoids (Fig 3B). The distribution of individual organoids for sphericity, shape factor and convexity on Day 5 are visualized with raincloud plots in Fig 3C to 3E. The UP organoids tended to be the most spherical (Fig 3C). On the other hand, the UK organoids had a lower shape factor and sphericity compared to the other PDTOs, suggesting that UK organoids form more cryptic structures (Fig 3D). The average convexity of UK organoids was also lower suggesting that there are more non-spherical cryptic organoids in the population consistent with the shape factor results (Fig 3E).

### Organoid growth is well-described by the Gompertz model, indicating an initial exponential growth phase

We leveraged the tracked imaging-based time series data introduced in the previous section to investigate the mathematical growth dynamics of PDTOs. Fig 1 gives an overview of the classical mathematical growth models employed in our analysis. Before fitting the models to the data, the number of live cells in each organoid was estimated from area measurements obtained via the NN image analysis (Section “Conversion from organoid area to cell number”). Then, for each individual organoid and each model, best-fit model parameters were computed by minimizing the least-squares error between the model prediction and the data (Section “Model fitting”). In total, seven datasets were analyzed, which included at least two biological replicates for each of the three patient organoid lines (Section “Patient tissue processing and organoid cultures”).

We first used the Bayesian Information Criterion (BIC), a common model selection tool [39], to assess the fit of each mathematical model to the observed data. The model with the lowest BIC is considered the most parsimonious model, taking into account that the mathematical models vary in complexity in terms of the number of model parameters (Section “Model selection”). Table 2 shows the average BIC across model fits for individual organoids in each of the UK/UP/US datasets. For the UP and US organoids, the Gompertz model fits the data the best, followed by the logistic model and then the von Bertalanffy model with exponent *γ* = 3/4 (Section “Mathematical models”). For the UK organoids, the Gompertz and logistic models show similar fit quality, and each model fits the data significantly better than the von Bertalanffy models. For all datasets, the models of constrained growth (Gompertz, logistic and von Bertalanffy) outperform the models of unconstrained growth (exponential, power law), indicating that most organoids show signs of reaching a growth plateau during the experiments.

**Table 2:** Model comparison results using BIC.

	Exp	PL 1/2	PL 2/3	PL 3/4	Gomp	Log	vB 1/2	vB 2/3	vB 3/4
UK–1	12.50	20.25	15.08	11.57	-20.90	**-21.09**	15.92	7.96	-0.74
UK–2	12.89	22.10	17.36	14.18	-20.53	**-21.11**	19.04	10.24	1.51
UP–1	20.91	18.49	16.50	16.51	**-16.75**	-10.75	5.45	-3.05	-7.91
UP–2	23.71	21.16	16.92	17.06	**-18.54**	-13.17	14.32	1.50	-4.71
US–1	12.66	11.70	11.81	10.94	**-18.55**	-15.75	1.20	-6.57	-9.55
US–2	14.66	12.76	12.25	12.06	**-7.41**	-5.96	3.51	-1.79	-3.82
US–3	14.30	13.81	11.99	12.32	**-15.44**	-13.06	-2.56	-7.37	-10.08

Average BIC obtained by fitting the mathematical models (Section “Mathematical models”) to each individual organoid in the UK/UP/US datasets (Section “Model selection”). The lowest BIC indicates the most parsimonious model, i.e. the best-fit model taking into account that the models vary in complexity in terms of the number of model parameters. The models considered are the exponential model (Exp), power law model (PL) with exponents *γ* ∈ {1/2, 2/3, 3/4}, Gompertz model (Gomp), logistic model (Log) and von Bertalanffy model (vB) with exponents *γ* ∈ {1/2, 2/3, 3/4}. The best-fit model for each dataset is indicated by bold.

In Table 3, we also compare the fit of the Gompertz, logistic and von Bertalanffy models using a simpler metric of model fit quality, which can be interpreted as the average percentage error between the model prediction and the observed data (Section “Model selection”). The model ranking using this simpler metric is consistent with the BIC ranking, with the Gompertz model performing the best overall. Table 3 furthermore shows model fitting errors relative to the Gompertz model error, which reveals that the difference between the Gompertz and von Bertalanffy models is more pronounced for the UK organoids than for the UP and US organoids. This suggests inter-patient heterogeneity in the organoid growth dynamics, which is further explored in the following sections. A more detailed comparison of fitting errors for individual organoids shows that for the UK datasets, the Gompertz and logistic models have essentially the same fitting error for almost all organoids (S3 Fig). Furthermore, many organoids in all datasets are well-fit by the logistic and von Bertalanffy models, and for some organoids the fit is even better than for the Gompertz model. However, a greater number of organoids is better fit by the Gompertz model, which results in it being the best-fit model overall.

**Table 3:** Model comparison results using normalized fitting error.

	Gompertz	Logistic	vB 1/2	vB 2/3	vB 3/4
UK–1	**0.0309**	0.0314	0.3149	0.1562	0.0975
		*1.0157*	*10.1822*	*5.0509*	*3.1521*
UK–2	**0.0237**	0.0241	0.3600	0.1766	0.1053
		*1.0194*	*15.2229*	*7.4683*	*4.4512*
UP–1	**0.0317**	0.0440	0.1605	0.0859	0.0622
		*1.3855*	*5.0545*	*2.7071*	*1.9601*
UP–2	**0.0201**	0.0323	0.1554	0.0681	0.0445
		*1.6033*	*7.7158*	*3.3823*	*2.2114*
US–1	**0.0334**	0.0376	0.1515	0.0926	0.0713
		*1.1251*	*4.5383*	*2.7742*	*2.1363*
US–2	**0.1011**	0.1287	0.1789	0.1397	0.1252
		*1.2735*	*1.7698*	*1.3823*	*1.2388*
US–3	**0.0490**	0.063	0.1449	0.0942	0.0767
		*1.2858*	*2.9587*	*1.9233*	*1.5662*

Mean normalized fitting error for the Gompertz, logistic and von Bertalanffy (vB) models with exponents *γ* ∈ {1/2, 2/3, 3/4} across individual organoids in the UK/UP/US datasets (Section “Model selection”). The normalized fitting error can be interpreted as the percentage error in the estimation. The best-fit model for each dataset is indicated by bold. The number in italics shows the mean normalized error relative to the Gompertz model error.

To further support our findings, we performed the same analysis on a different dataset (US–GFP dataset; Section “Patient tissue processing and organoid cultures”) [35]. This dataset is small but has the advantages of an extra datapoint per organoid (Days 0, 1, 3, 6) and direct measurements of the number of live cells in each organoid. In short, the Gompertz model continues to be the best-fit model for this dataset, followed by the logistic and von Bertalanffy models (S2 Table). For the two US-GFP biological replicates, the mean normalized fitting errors of the von Bertalanffy model with *γ* = 3/4 were 25.9% (Plate 1) and 37.6% (Plate 2) larger, respectively, than for the Gompertz model (S3 Table). This is comparable to the relative errors of the von Bertalanffy and Gompertz models for the US–2 and US–3 datasets (Table 3). It should be noted that the model fit difference between the Gompertz and von Bertalanffy models is even more pronounced for the UK and UP organoids according to Tables 2 and 3.

### Distribution of the initial exponential growth rate is lognormal and indicates significant intrapatient heterogeneity

An inspection of the growth trajectories of individual organoids reveals significant heterogeneity in the growth dynamics, even for multiple organoids from the same patient. To investigate this intrapatient heterogeneity, we analyzed how the estimated values of the Gompertz model parameters *a* and *b* for individual organoids are distributed within each dataset. The Gompertz model parameter *a* captures the initial exponential growth rate of the organoid, while the parameter *b* captures how quickly organoid growth decays over time (Section “Gompertz model”). Under the Gompertz model, each organoid eventually reaches a so-called “carrying capacity” *K*, which is the predicted final size of the organoid (Fig 1 and Section “Gompertz model”).

**Fig 4:**
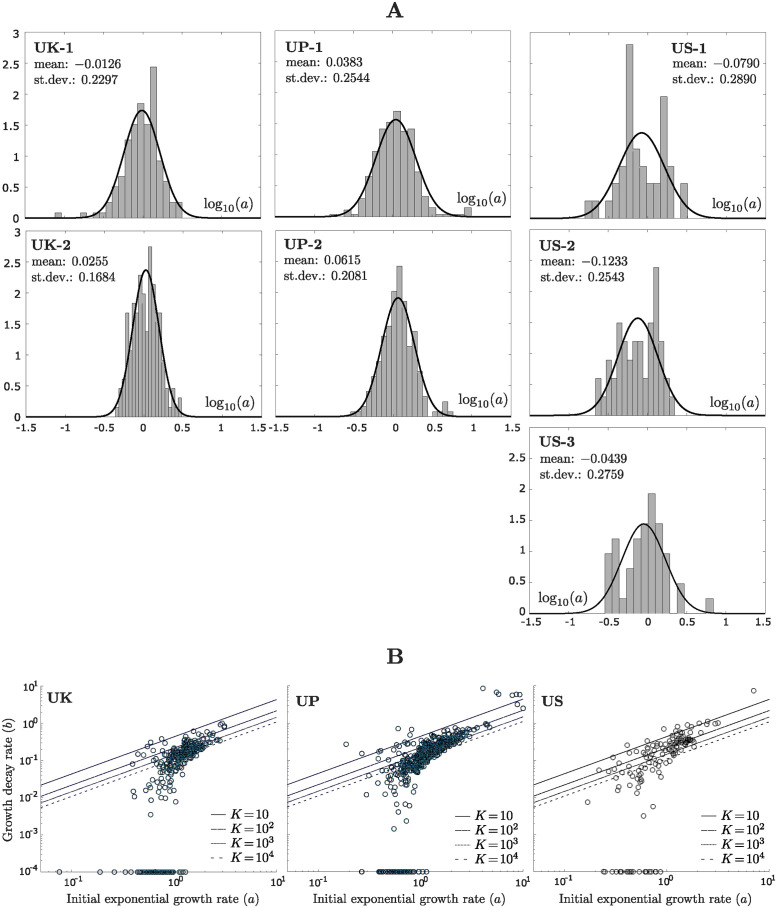
Intrapatient heterogeneity in tumor organoid growth. A: Distribution of log_10_(*a*) within the UK/UP/US datasets, where log_10_(*a*) denotes the logarithm of *a* with base 10. For each organoid in each dataset, the initial exponential growth rate *a* of the organoid was estimated using the Gompertz model (Sections “Gompertz model” and “Model fitting”). Each panel shows how the estimated values of *a* are distributed across individual organoids within the indicated dataset, under a logarithmic transformation of *a*. The logarithmic transformation is applied since the estimated values of *a* vary across an order of magnitude within each dataset. For each dataset, the distribution of log_10_(*a*) is consistent with a normal distribution, meaning that we fail to reject the null hypothesis of normality under a Kolmogorov-Smirnov statistical test at the 5% significance level. B: Distribution of the Gompertz parameters (*a*, *b*) within the UK/UP/US datasets, shown on a logarithmic scale. The different datasets for each patient have been combined. For each organoid in each dataset, the initial exponential growth rate *a* and the rate of growth decay *b* were estimated using the Gompertz model (Sections “Gompertz model” and “Model fitting”). Each dot in each panel represents a single organoid, where the horizontal position of the dot indicates the value of *a* for that organoid and the vertical position indicates the value of *b*. When fitting the Gompertz model to individual organoids, we set *b* = 10^−4^ as the smallest possible value for *b* and treat it as effectively zero. Organoids with *b* = 10^−4^ are referred to as “exponential organoids”, while the remaining organoids are referred to as “nonexponential”. The exponential organoids are all situated on the horizontal axis and their position on the axis represents their rate of exponential growth. The slanted lines indicate carrying capacities of *K* = 10, 10^2^, 10^3^, 10^4^, where the carrying capacity is the predicted final size of the organoid under the Gompertz model (Fig 1). All organoids falling on the same line share the indicated carrying capacity. Organoids falling below the lowest line are predicted to have a final size above 10^4^ cells.

Fig 4A shows that for each of the UK/UP/US datasets, the distribution of log_10_(*a*) across individual organoids is consistent with a normal distribution, where log_10_(*a*) denotes the logarithm of *a* with base 10. The same is true if we combine the datasets for each patient before testing for normality (S4 Fig). This is confirmed by Kolmogorov-Smirnov statistical tests at the 5% significance level. We applied a logarithmic transformation to *a* before assessing normality since the estimated values of *a* vary across an order of magnitude within each dataset, which indicates significant intrapatient heterogeneity in the values of *a*. We performed the same analysis for the US–GFP dataset and continued to observe a lognormal distribution for *a*, both for the individual replicates and the overall dataset (S5 Fig).

We were next interested in examining the two-dimensional distribution of (*a*, *b*) for the three patient samples, shown on a logarithmic scale in Fig 4B. We first note that for many organoids in each dataset, the growth decay parameter *b* is effectively zero (*b* = 10^−4^), in which case the organoid grows at exponential rate *a* through the end of the experiment. We will refer to these organoids as “exponential” and the remaining organoids as “nonexponential”. The proportion of exponential organoids varies between the patient samples (24.8% for UK, 10.7% for UP, 16.2% for US), but the growth rates of these organoids are clearly smaller for US than for UK and UP (Figs 4B and S8A). We then recall that under the Gompertz model, the carrying capacity of an organoid with parameters *a* and *b* is given by *K* = *e*^*a*/*b*^ (Sections “Mathematical models” and “Variable dormancy time”). Thus, organoids that share a common ratio *a*/*b* = *k* between the parameters *a* and *b* have the same carrying capacity. In Fig 4B, we have drawn slanted lines along which the carrying capacity is *K* = 10, 10^2^, 10^3^, 10^4^. We note that for all the patient samples, several nonexponential organoids are estimated to have carrying capacities over 10,000 cells. For these organoids, the estimated carrying capacity is significantly larger than the observed final size on Day 5 (S6 Fig), indicating that only a small part of the overall growth trajectory has been observed in the experiment. We finally note that the distribution of nonexponential organoids is more concentrated for the UK and UP organoids than the US organoids, indicating greater heterogeneity in organoid growth for the US organoids. We discuss inter-patient differences further in the following section.

For the nonexponential organoids, the Gompertz parameters *a* and *b* display a high degree of correlation (0.77 for UK, 0.76 for UP and 0.85 for US). This indicates a strong linear relationship between *a* and *b*, in the sense that smaller values of *a* tend to coincide with smaller values of *b*. Vaghi et al. [40] recently modeled the growth of three animal models of breast and lung cancer using the Gompertz model. For each animal model, they observed an almost exact linear relationship *a* = *kb* between *a* and *b*, indicating a common carrying capacity for all animals transplanted with the same cancer cells. This and similar evidence from the literature [19, 41–43] led the authors to model their data using a reduced Gompertz model, where the carrying capacity was assumed fixed among all animals with the same cancer. For our organoid datasets, even though *a* and *b* are highly correlated, they do not appear to obey an exact linear relationship leading to a common carrying capacity within each patient sample. This again highlights the level of heterogeneity in organoid growth, even for organoids derived from the same patient and grown in the same environment.

To ensure that the observed intrapatient heterogeneity in *a* and *K* is not caused by differences in the positions of organoids within the experimental wells, we verified that there is no clear relationship between the values of *a* and *K* and the distance of the organoid from the center of the well within any of the patient samples, see S7 Fig.

### Differences in growth characteristics between patients can be explained by differences in growth rate and dormancy time

**Fig 5:**
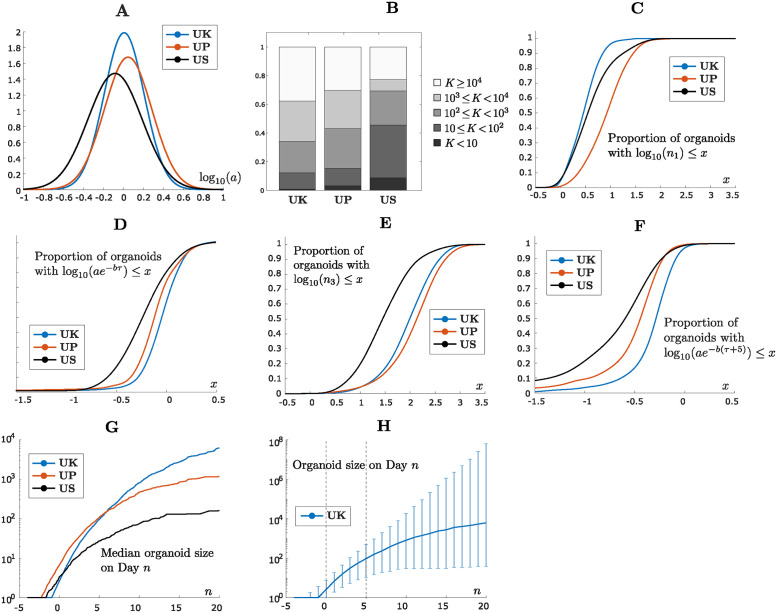
Interpatient heterogeneity in tumor organoid growth. A: Comparison of the distributions of log_10_(*a*) between the different patient samples, where the datasets for each patient have been combined. B: Comparison of the distributions of carrying capacities between the different patient samples. Only nonexponential organoids are considered (*b* > 10^−4^). For each organoid in each dataset, the carrying capacity *K* = *e*^*a*/*b*^ of the organoid was estimated using the Gompertz model (Sections “Gompertz model” and “Model fitting”). The columns indicate for each patient the proportion of organoids falling within each category *K* < 10, 10 ≤ *K* < 10^2^, 10^2^ ≤ *K* < 10^3^, 10^3^ ≤ *K* < 10^4^ and *K* ≥ 10^4^. C: Comparison of the distributions of log_10_(*n*_1_) between the different patient organoids, where the datasets for each patient have been combined, and *n*_1_ is the observed size of the organoid on Day 0. Here, log_10_(*n*_1_) denotes the logarithm of *n*_1_ with base 10. For each patient sample, the graph of the cumulative distribution function (CDF) of log_10_(*n*_1_) is shown, which gives for each value of *x* the proportion of organoids satisfying log_10_(*n*_1_) ≤ *x*. The CDF has been estimated using the *ksdensity* function in MATLAB. The fact that the UK organoid graph lies farthest to the left means that UK organoids are the smallest on average on Day 0, while the UP organoids are the largest on average on Day 0. D: Comparison of the distributions of log_10_(*ae*^−*bτ*^) between the different patient organoids, where *ae*^−*bτ*^ is the growth rate of the organoid on Day 0 according to the Gompertz model. The UK organoids have the largest growth rates on average on Day 0, while the US organoids have the smallest growth rates on average. E: Comparison of the distributions of log_10_(*n*_3_) between the different patient organoids, where *n*_3_ denotes the observed size of the organoid on Day 5. The UP organoids are slightly larger than the UK organoids overall on Day 5, and both the UP and UK organoids are significantly larger than the US organoids overall. F: Comparison of the distributions of log_10_(*ae*^−*b*(*τ*+5)^) between the different patient organoids, where *ae*^−*b*(*τ*+5)^ is the growth rate of the organoid on Day 5 according to the Gompertz model. The UK organoids have the largest growth rates on average on Day 5. G: Median organoid size projected to Day 20 for each patient sample. To generate the curves, we sampled 100,000 sets of Gompertz parameters (*a*, *b*) from the observed parameter distributions for each patient, and computed the medians of the sampled curves. H: UK organoid size projected to Day 20 with error bars. The limits of the error bars represent the 5th and 95th percentile, respectively, of the 100,000 sampled curves from part G.

We finally used the Gompertz modeling perspective to gain insights into the differences in growth dynamics between the different patient organoids. In Fig 5A, we compare the distributions of log_10_(*a*) for the UK, UP and US organoids. The US organoid distribution is significantly different from the UK and UP distributions according to a Kolmogorov-Smirnov test, whereas the difference between UK and UP is not significant at the 5% level when a Bonferroni correction is applied (*p*-values 1.3 ⋅ 10^−4^ for UK vs. US, 1.7 ⋅ 10^−5^ for UP vs. US and 0.039 for UK vs. UP). The US organoid distribution is the widest and has the lowest average growth rate. However, for the most part, the three distributions overlap, indicating that the level of intrapatient heterogeneity in growth rates exceeds the level of interpatient heterogeneity. In particular, even though the US organoids grow the slowest on average, it is not the case that all US organoids have smaller growth rates than all UK and UP organoids.

In Fig 5B, we show for each patient sample how the predicted carrying capacities of nonexponential organoids are distributed between the size categories of Fig 4B. A more detailed distribution is shown in S8B Fig. The carrying capacities of UK organoids are generally larger than for the UP organoids, while the US organoids have significantly smaller carrying capacities overall. In our formulation of the Gompertz model, we have assumed that a seeded single cell starts growing into an organoid on Day −*τ*, where 0 ≤ *τ* ≤ 4 is allowed to vary between organoids (Section “Variable dormancy time”). This reflects the fact that after seeding, individual cells may stay dormant for varying amounts of time while adjusting to the new environment. If a seeded cell starts growing on Day −*τ* with an initial exponential growth rate *a*, then under the Gompertz model, the growth rate has decreased to *ae*^−*bτ*^ by Day 0 (Section “Gompertz model”). Interestingly, the UK organoids are the smallest on average on Day 0 (Fig 5C), even smaller than the US organoids, yet they have the largest growth rates on Day 0 according to the Gompertz model fits (Fig 5D). On Day 5, the UK organoids have grown to be significantly larger than the US organoids, and they have almost caught up in size with the UP organoids (Fig 5E). In addition, the UK organoids still have the largest growth rates *ae*^−*b*(*τ*+5)^ on Day 5 (Fig 5F). As a result, the UK organoids are eventually predicted to become larger than the UP organoids overall, as is indicated by the distributions of carrying capacities for UK and UP organoids in Fig 5B.

One useful application of our mathematical modeling is that it enables prediction of organoid growth beyond the final observed experimental date. In Fig 5G, we show median projected growth trajectories for the three patient samples up until Day 20. Consistent with previous insights, the median UK organoid starts growing later than the other two organoids, and the UK organoid is smaller than the UP organoid during the experimentally observed period. On Day 20, the median UK organoid is larger than the UP organoid, which is in turn significantly larger than the median US organoid. In Fig 5H, we show the projected growth trajectory for the UK organoid with error estimates, which capture both the intrapatient heterogeneity in the growth dynamics up until Day 5 and the increasing uncertainty in the projected trajectory as it extends further beyond the experimental dates.

To interpret our findings in a clinical context, it is important to note that the *in vitro* dynamics of organoid growth are significantly faster than the dynamics of tumor growth within a human patient. A direct comparison of time scales is difficult since under Gompertzian growth, for example, the tumor doubling time decreases as the tumor grows larger. However, to get a sense, we note that for the median UK organoid trajectory, the model-estimated doubling time is around 16 hours at the beginning of organoid growth, while it has decreased to 2.6 days by Day 10. In contrast, in a recent study of clinical data from 43 colorectal cancer patients, the median tumor doubling time was found to be 211 (112–404) days [44]. If we use a scaling factor of 211/2.6, then 20 days in the *in vitro* setting correspond to 4.4 years in a patient. We stress that this number is only an indication, as the doubling times depend on several factors such as the disease stage, and they are heterogeneous between patients. However, it is clear that understanding organoid growth over a time span of 20 days in the *in vitro* setting can potentially yield clinical insights on a much longer time scale.

## Discussion

Biological investigations using organoid models necessitate the application of 3D imaging techniques to examine dynamic organoid features. However, capturing multi-dimensional information (size, morphology, viability status) from a time series of images is difficult. By training and applying a NN on EFI projected images, we were able to automate the process, supporting multi-scale batch analysis with non-uniform phenotypic measurements. The ability to individually track organoids facilitates the monitoring of their temporal dynamics, introducing an additional layer of patient-specific features. Since PDTOs replicate inter- and intra-patient heterogeneity, it is important to understand their growth and morphological characteristics before exploring biological mechanisms and utilizing them as a platform for investigating drug effectiveness. In this work, we suggest an integrated experimental-computational method to explore PDTO growth by combining a high-throughput imaging-based platform with mathematical modeling. While our method was initially applied to CRC PDTOs, it can easily be adapted to different organoid types.

The integration of organoid imaging data with mathematical modeling has enabled us to gain quantitative insights into organoid growth dynamics and to predict their future growth. This integration can further be leveraged for drug discovery and to assist in selecting drugs when faced with drug resistance, or in identifying optimal combination therapies. Through our mathematical analysis, we found that organoid growth is well-described by the Gompertz model, which involves an initial exponential growth phase. Exponential growth entails that on average, a constant proportion of cells in the organoid divides over time, where divisions can nevertheless occur in an asynchronous and stochastic manner (Section “Exponential model”). The fact that the Gompertz model is preferred over the von Bertalanffy model indicates that at the very initial stages of organoid growth, cell divisions occur uniformly across the organoid, as opposed to being restricted to the outermost cell layer. We note that in our model formulation, seeded single cells are assumed to lay dormant for some period before starting to grow and form organoids (Section “Variable dormancy time”). When we say that the initial growth is exponential, we mean that it is exponential from the time the organoid starts growing. Alternative potential explanations of the initial growth dynamics are discussed below.

In further analysis of the Gompertz model fits, we observed significant intrapatient heterogeneity in the Gompertz model parameters *a*, the initial exponential growth rate, and *K*, the carrying capacity. The distribution of *a* amongst individual organoids was consistently lognormal [45–48], both across different datasets for the same patient and across the different patients. As for interpatient heterogeneity, the UK and UP organoids showed similar distributions for *a* and *K*, despite the UK organoids being the smallest on average on Day 0 and growing the fastest between Day 0 and Day 5. Our mathematical model suggests a simple explanation for this, which is that the UK organoids have longer dormancy times after seeding than the UP and US organoids. This is reflected in how the estimated values of the starting time parameter *τ* are distributed within each patient sample. The distribution of *τ* is skewed toward *τ* = 0 for the UK organoids, meaning that the organoids usually start growing close to Day 0, and toward *τ* = 4 for the UP organoids, meaning that the organoids usually start growing close to Day −4 (S9 Fig). For the US organoids, the distribution of *τ* more resembles a bimodal distribution, with small and large values of *τ* both being common. Overall, the US organoids showed quite distinct growth characteristics from the UK and UP organoids, and the estimated values of *a*, *K* and *τ* indicated an elavated level of heterogeneity in organoid growth compared to the UK and UP organoids.

The UK and UP organoids were established from advanced tumor tissues (stage 4A and 4, respectively) compared to US organoids (stage 2), potentially explaining why UK and UP organoids grow faster than US organoids (Table 1). In Burke et al. [44], they found that the median colon tumor growth per time period of 62 days was greater for more advanced tumors, supporting our *in vitro* PDTO findings. Additionally, the US organoid has a KRAS G12A mutation and a high tumor mutational burden, which may contribute to the observed heterogeneity in US organoid growth through dysregulation of cell proliferation. It is unclear to what extent the carrying capacities of organoids predict the carrying capacities of the tumors themselves. Organoids are good *in vitro* models for patients’ tumors but the physiological environment is not the same as *in vivo*. Tumor cells in tissue interact with a variety of stromal cells (fibroblasts, endothelial cells, immune cells, etc.) and tumor growth can be affected by other microenvironmental factors such as tissue stiffness, oxygen gradient and nutritional supports. Further studies are needed to directly correlate the carrying capacities of organoids with carrying capacities of patient tumors. Overall, molecular differences between patients play a significant role in shaping organoid growth, morphology and response to drugs. Subsequent work will examine connections between the current growth model and individual patient characteristics, aiming to explore personalized therapeutic strategies.

### Limitations

We acknowledge uncertainty in the estimation of growth parameters and in the selection of the most appropriate growth model due to a limited number of datapoints per organoid. Nevertheless, our results show that the Gompertz model is useful for understanding the basic mathematical properties of organoid growth, and these results are consistent with previous evidence for the relevance of the Gompertz model for tumor growth. We note that three-dimensional imaging data indicates that some of our PDTOs become hollow, cystic-like, as they grow larger. Since the Gompertz model is nonspatial and empirical in nature, this does not preclude its application to the data. However, in future work we plan to develop growth models which take the spatial characteristics of organoid growth into account, while remaining simple enough to be applicable to the high-throughput setting. We also note that the mathematical models employed in this study assume that each organoid can be treated as a homogeneous cell population, while in reality, each organoid is likely composed of a mix of different cell types, both stem and differentiated cells. In future work, we plan to explore models which incorporate intra-organoid heterogeneity in cell type, since the observed variability in growth profiles is likely driven in part by heterogeneity in cell type composition. The new growth models could potentially leverage the morphology data described in Section “Patient-specific organoid size and morphological changes can be measured by AI-driven image analysis with individual organoid tracking”, which we have not incorporated into the mathematical models of the present study.

Furthermore, in our model formulation, we have assumed that after seeding, a cell may remain dormant for some time before starting to grow and form an organoid, and this time is allowed to vary between different organoids. We have shown that this is sufficient to explain the fact that UK organoids are significantly smaller than UP organoids on Day 0 while growing faster between Days 0 and 5. However, there are several other potential explanations for this behavior. For example, organoid growth at the initial stages may be partly driven by cell aggregation, the level of which may differ between the UK and UP organoids. It is also possible that the growth dynamics at very low cell densities, before cell-cell interactions become a significant factor, deviate from the overall dynamics. For example, an in vitro culture of BT-474 luminal B breast cancer cells was recently observed to display an Allee effect, under which population growth is significantly slower at very low densities [49].

We finally note that in this work, we have employed a data filtering process in part to alleviate segmentation errors made by the NN (Section “Data filtering”). In future work, we plan to continue to develop and improve the NN image analysis, for example by generating training data involving more patient samples. In addition, we have in this work only considered alive organoids that grow between every pair of time points, whereas modeling drug screening data may require extending the models to allow for nonmonotone growth.

### Conclusions

As far as we are aware, this is the first work to use simple tumor growth models to gain insights into the growth characteristics of patient-derived tumor organoids and to highlight both intra- and interpatient differences in the dynamics. Understanding these differences promotes the development of model-driven precision medicine, since knowing the growth dynamics of the different patient organoids in the untreated condition sets the baseline for understanding how the same organoids are affected by drug treatment. Deriving translational value from the combination of mathematical modeling with organoid growth experiments and organoid drug screens will require further development both on the experimental and mathematical modeling side. Ultimately, we hope to integrate mathematical modeling with high-throughput drug screenings to facilitate drug discovery, drug testing and personalized treatment optimization.

## Supporting information

S1 Text Supplementary text. (PDF)

S1 Fig Graphical abstract. Image was generated using the BioRender software.
(TIF)

S2 Fig Live-cell imaging. A: Live cell imaging of US-GFP organoid. Cell division events are highlighted. Single Z plane at the center of organoid was imaged over time (every 20 minutes for 12 hours). Time stamp, hours: minutes: seconds. Scale bar, 20 micrometer. (S4 Video) B: Immunostaining of US organoid shows the actively proliferating cells (Green-labeled). Ki67 (Green): Cell proliferation, E-cadherin (Red): Cell junction, DAPI (Blue): Cell nuclei.
(TIF)

S3 Fig Comparison of normalized fitting errors between Gompertz, logistic and von Bertalanffy models. A: Comparison of normalized fitting errors (Section “Model selection”) individual organoids in the UK/UP/US datasets between the Gompertz and logistic models. Each dot represents a single organoid. B: Comparison of normalized fitting errors between the Gompertz model and the von Bertalanffy model with *γ* = 3/4.
(PDF)

S4 Fig Distribution of log_10_(*a*) across individual organoids in the UK/UP/US datasets. The different datasets for each patient have been combined. The mean and standard deviation of each distribution are shown. For each patient, the distribution of log_10_(*a*) is consistent with a normal distribution according to a Kolmogorov-Smirnov test at the 5% significance level, meaning that we fail to reject the null hypopthesis of normality.
(PDF)

S5 Fig Distribution of log_10_(*a*) across individual organoids on each experimental plate (left two panels) and overall (rightmost panel) for the US–GFP dataset. The mean and standard deviation of each distribution are shown. For each plate individually and for the two plates combined, the distribution of log_10_(*a*) is consistent with a normal distribution according to a Kolmogorov-Smirnov test at the 5% significance level.
(PDF)

S6 Fig Final observed organoid size on Day 5 vs. the carrying capacity predicted by the Gompertz model. The datasets for each patient have been combined. Each dot represents a single organoid and only nonexponential organoids are considered (*b* > 10^−4^). Carrying capacities above 10^10^ cells are set to 10^10^.
(PDF)

S7 Fig Intrapatient heterogeneity in organoid growth is not driven by position within experimental wells. A: Comparison between the distance *r* of an organoid from the center of the experimental well and the initial exponential growth rate *a* of the organoid. Each dot represents a single organoid. The datasets for each patient have been combined. For each patient, a best-fit line is shown. The slope of the line cannot be distinguished from zero at the 5% significance level for any of the patient samples when a Bonferroni correction is applied. B: Comparison between the distance *r* of an organoid from the center of the experimental well and its estimated carrying capacity *K* according to the Gompertz model. Only nonexponential organoids are considered (*b* > 10^−4^), and carrying capacities above 10^10^ cells are set to 10^10^. For each patient, a best-fit line is shown. The slope of the line cannot be distinguished from zero at the 5% significance level for any of the patient samples, whether or not a Bonferroni correction is applied.
(PDF)

S8 Fig Interpatient heterogeneity in initial growth rate and carrying capacity. A: Comparison of the distributions of log_10_(*a*) between the different patient samples, where only exponential organoids are considered (*b* = 10^−4^), and the datasets for each patient have been combined. For each patient sample, the graph of the cumulative distribution function (CDF) of log_10_(*a*) is shown, which gives for each value of *x* the proportion of organoids satisfying log_10_(*a*) ≤ *x*. B: Comparison of the distributions of carrying capacities between the different patient samples, where only nonexponential organoids are considered (*b* > 10^−4^). For each patient sample, the graph of the CDF of log_10_(*K*) is shown.
(PDF)

S9 Fig Distribution of *τ* across individual organoids for the different patient samples, where the datasets for each patient have been combined. In our model, it is assumed that each single cell starts growing into an organoid on Day −*τ*, where the parameter *τ* is allowed to vary between organoids. For each organoid in each dataset, the value of the parameter *τ* was estimated using the Gompertz model (Sections “Gompertz model” and “Model fitting”), and each panel shows how the estimated values of *τ* are distributed across the individual organoids for each patient. The distribution of *τ* is skewed toward *τ* = 0 for UK organoids and toward *τ* = 4 for UP organoids. For US organoids, the distribution of *τ* more resembles a bimodal distribution, where both small and large values of *τ* are common.
(PDF)

S1 Table The results of each data filtering step described in Section “Data filtering”. (PDF)

S2 Table Average BIC obtained by fitting each mathematical model to each individual organoid in the US–GFP dataset. The best-fit model for each plate is indicated by bold.
(PDF)

S3 Table Mean normalized error obtained by fitting the Gompertz, logistic and von Bertalanffy (vB) models to each individual organoid in the US–GFP dataset. The best-fit model for each plate is indicated by bold.
(PDF)

S1 Video Live imaging of US-GFP organoids. Condensed chromosomes and dividing cells are shown in the movie.
(AVI)

S2 Video NN processing and tracking of UK organoids from Day 0 to Day 5. Live organoids were labeled with blue and green lines to show the tracking of organoids over time.
(AVI)

S3 Video NN processing and tracking of UP organoids from Day 0 to Day 5. Live organoids were labeled with blue and green lines to show the tracking of organoids over time.
(AVI)

S4 Video NN processing and tracking of US organoids from Day 0 to Day 5. Live organoids were labeled with blue and green lines to show the tracking of organoids over time.
(AVI)
