# Supplementary material for: Understanding patient-derived tumor organoid growth through an integrated imaging and mathematical modeling framework
Source: PLoS Comput Biol. 2024 Aug 2;20(8):e1012256. doi: 10.1371/journal.pcbi.1012256 (PMC11324155; doi:10.1371/journal.pcbi.1012256)
Supplement: S1 Text — (PDF) [file pcbi.1012256.s001.pdf]

Supplementary Text

Understanding patient-derived tumor organoid growth through an integrated imaging and mathematical modeling framework

Einar Bjarki Gunnarsson, Seungil Kim, Brandon Choi, J. Karl Schmid, Karn Kaura, Heinz-Josef Lenz, Shannon M. Mumenthaler, Jasmine Foo

1 Previous investigations of tumor growth laws

The use of simple mathematical growth models like the exponential, power law, Gompertz, logistic and von Bertalanffy models to investigate the dynamics of tumor growth goes back several decades. A seminal paper by Laird [1] applied the Gompertz model, which was developed in the 19th century to describe human mortality curves [2], to the growth of 19 animal tumor models. The Gompertz model has since become a popular tumor growth model, with notable examples including frequent applications of Norton and colleagues to human breast cancer data [3, 4, 5]. Marusic et al. [6, 7] fit a multitude of growth models to tumor spheroid and mouse data, and they found that many models, including the Gompertz model, fit the data well, while the logistic and von Bertalanffy model could not fit the data. The Gompertz model was also found to outperform the logistic and von Bertalanffy models for MCF-7 breast cancer spheroids [8], and it was more recently shown to perform well on several in vivo experimental systems of breast and lung cancer [9, 10], as well as a large dataset of patients undergoing chemo- or immunotherapy [11]. An exponential-linear model, which was motivated by Gompertz growth in the absence of the tumor reaching a growth plateau [12], has furthermore been used successfully for example in [12, 9, 13].

Despite the proven relevance of the Gompertz model for tumor growth, it is not always the most appropriate growth model. Vaidya and Alexandro [14] found that the logistic equation fit data from seven lung cancer patients best, whereas seven out of ten mice with induced sarcoma were best fit by the von Bertalanffy model. Talkington and Durrett [15] found that patient data for breast and liver cancer was best described by exponential growth, while a 2/3 power law model was the most appropriate for two neurological cancers. The 2/3 power

law model is a special case of the classical von Bertalanffy model, where proliferation is constrained to the tumor surface but no growth plateau is reached (Section "Mathematical models" of the main text). For patient data involving Grade II gliomas [16], the tumor diameter was found to grow linearly over time, which is consistent with 2/3 power law growth. A 3/4 version of the von Bertalanffy model was proposed by West et al. [17] as a general model of ontogenetic growth based on allometric principles. A study by Sarapata and de Pillis [18] fit the exponential, power law, logistic, Gompertz and von Bertalanffy models to a mix of in vitro and in vivo data for ten different cancer types. They found that the power law model fit the data best for six out of ten cancer types, the logistic for three and the Gompertz for one, although they subsequently discouraged the use of the power law model both for biological reasons and due to parameter fitting sensitivity. Many other investigations along these lines have been carried out, as is discussed for example in the review by Rodrigeuz-Brenes et al. [19].

Overall, prior work indicates that the appropriate tumor growth model is context-dependent. Gerlee [20] refers to a "model muddle", and he argues for the use of the von Bertalanffy model over the Gompertz and logistic models, since it has the advantage of being biologically motivated. It should be noted that the preferability of one growth model over another can be influenced by the chosen parameter fitting procedure, as is pointed out for example in [7, 9].

2 Filtering of image analysis results

When reviewing the results of the automated NN image analysis (Section "Confocal 3D live cell imaging and quantitative image analysis" of the main text), we noticed some segmentation errors made by the NN. Specifically, the NN sometimes identified two organoids which were close to one another or overlapped in the 2D projected image as a single organoid. In addition, it sometimes identified a single large organoid as two or more smaller organoids. We constructed a data filter designed to detect such merging and splitting events, using the fact that the NN identifies both organoids that can be tracked through all time points of the experiment as well as organoids that cannot be tracked.

The basic idea of the filter is to check for each tracked organoid at each time point how

many untracked organoids are within a certain distance from it. If a tracked organoid has an untracked organoid in its vicinity at a certain time point, but no untracked organoid in its vicinity at the next time point, this is a sign that the untracked organoid may have merged with the tracked organoid. On the other hand, if a tracked organoid has no untracked organoid in its vicinity at a certain time point, but an untracked organoid appears in its vicinity at the next time point, this is a sign that the tracked organoid may have been split into two organoids by the NN. Following this logic, the simplest way to define a data filter is to only accept tracked organoids which have no untracked organoids in their vicinities at any time point. However, it is reasonable to allow merging or splitting events which are insignificant compared to the size of the tracked organoid in question. We therefore defined a filter which allows untracked organoids to be in the vicinity of a tracked organoid, but with restrictions on the number and total size of these untracked organoids. We furthermore filtered out tracked organoids reducing in size by more than 50% between Days 3 and 5 and any tracked organoids in their vicinities. The filtering process is outlined in Algorithm 1.

Algorithm 1: Data filter to remove merging and splitting events

---

1 For each tracked organoid and each time point, identify all untracked organoids whose centers of mass (identified by the NN) are within a certain distance from the center of mass of the tracked organoid. The distance is set to 75 μm on Day 0 and 50 μm on Days 3 and 5.

2 For each tracked organoid and each time point, compute the number and total area of the untracked organoids in its vicinity as identified by Step 1.

3 For a given tracked organoid, let A_1, A_2, A_3 denote its area on Days 0, 3 and 5, respectively, let u_1, u_2, u_3 denote the number of untracked organoids in its vicinity, and let a_1, a_2, a_3 denote their total areas at the different time points. We only accept the tracked organoid if all of the following conditions are met:

   (i) $u_1 \leq 2$ and ($a_1 \leq 300$ μm^2 or $a_1/A_1 \leq 0.1$)

   (ii) $u_i \leq 1$ and ($a_i \leq 300$ μm^2 or $a_i/A_i \leq 0.1$) for $i = 2, 3$.

4 For each tracked organoid and each time point, identify all tracked organoids whose centers of mass are within 50 μm from the center of mass of the tracked organoid.

5 Remove any tracked organoid which declines by 50% in size between the second and third time point, as well as any tracked organoid in its vicinity.

To evaluate the performance of the data filter, we manually reviewed 2D projected images from four wells for each of the patient organoids (twelve wells in total). For each tracked organoid in each well, we assessed visually whether the organoid had been merged with a

nearby organoid by the NN or it had been split into two or more organoids by the NN. We then manually labeled each organoid according to the six categories shown in Table 1. Labels A and B were used for organoids with slight or no merging or splitting issues, and labels X and Y were used for organoids affected by significant merging or splitting events. Label C was used for boundary cases and label Z was used for other issues. Results of the labeling for the twelve wells are shown in Table 2. The distribution of organoids between categories is shown both for organoids accepted by the filter and for organoids rejected by the filter. Table 2 shows that most organoids accepted by the filter either have slight or no merging or splitting issues (labels A and B), while most organoids rejected by the filter have significant issues (labels X and Y). Table 3 shows the precision of the data filter for the reviewed wells, which is the percentage of accepted organoids labeled as having slight or no issues. The precision is high across wells, especially if the boundary cases (label C) are regarded as being acceptable. This indicates that the data filter we created is successful in removing most of the merging and splitting errors made the NN image analysis.

3 Fitting the von Bertalanffy model with $\gamma > 3/4$

In Section "Organoid growth is well-described by the Gompertz model, indicating an initial exponential growth phase" of the main text, we only considered the von Bertalanffy model with exponents $\gamma \in \{1/2, 2/3, 3/4\}$, of which $\gamma = 2/3$ and $\gamma = 3/4$ are established choices for tumor growth modeling. We note here that if γ is increased above 3/4, the fit quality of the von Bertalanffy model becomes progressively better as the exponent increases, as is demonstrated in Table 4. For $\gamma = 0.99$ in particular, the fitting error is almost as small as for the Gompertz model for all datasets. This is due to the fact that the von Bertalanffy differential equation becomes an approximation of the Gompertz differential equation as $\gamma \to 1$, given the appropriate reparametrization, as we discussed in Section "Relationship between the models" of the main text. To confirm this theoretical insight, we compared the fitted growth curves produced by the Gompertz and von Bertalanffy models, and observed that the growth curves approach one another as $\gamma \to 1$ (Table 5).

4 Alternative conversion from organoid area to organoid volume

In the main text, we converted the area measurement obtained via the NN image analysis to a volume estimate for each individual organoid. For the conversion, we assumed that each organoid is an ellipsoid and that the third axis is the geometric mean of the other two axes, $c = \sqrt{ab}$. Then, the volume of the organoid can be written in terms of its area as

$$V = \frac{4}{3}\pi abc = \frac{4}{3\sqrt{\pi}}A^{3/2}.$$

However, the NN image analysis also returns morphology measurements which can be used to estimate a and b for each individual organoid. To check how sensitive our model fitting results are to the particular method chosen to obtain volume estimates, we now compare the model fit quality of the mathematical models using the following volume estimates:

(i) $V = \frac{4}{3}\pi a^2 b$ (the third axis is equal to the first axis).

(ii) $V = \frac{4}{3}\pi ab^2$ (the third axis is equal to the second axis).

The results, shown in Tables 6 and 7, mimic the model fitting results in Tables 2 and 3 of the main text.

5 Assessing model fit quality using logarithmized data

In the main text, we fit each growth model to each individual organoid by minimizing the least squares error between the model prediction and the data (Section "Model fitting" of the main text). To check whether our model fitting results are sensitive to the chosen parameter fitting procedure, we now reconduct the model fitting under a logarithmic transformation of the model and the data,

$$(\hat{\boldsymbol{\theta}}_{\text{alt}}, \hat{\tau}_{\text{alt}}) := \text{argmin}_{\boldsymbol{\theta}} \textstyle\sum_{i=1}^{k} \left(\log(n_i) - \log(N(\tau + t_i; \boldsymbol{\theta}, \tau))\right)^2. \quad (20)$$

This is the maximum likelihood estimate for the statistical model

$$\log(n_i) = \log(N(\tau + t_i; \boldsymbol{\theta}, \tau)) + \varepsilon_i \quad \text{i.e.} \quad n_i = N(\tau + t_i; \boldsymbol{\theta}, \tau)\exp(\varepsilon_i), \tag{21}$$

where $\varepsilon_1, \ldots, \varepsilon_k$ are independent and identically distributed $N(0, \sigma^2)$ random variables with $\sigma^2 > 0$. Note that $\exp(\varepsilon_i) \approx 1 + \varepsilon_i$ for small $\varepsilon_i > 0$. Thus, the difference between (18) in the main text and (21) here is that in (18), the error term is independent of organoid size, while in (21), the error scales with the size. The results are shown in Tables 8 and 9. As in Section "Organoid growth is well-described by the Gompertz model, indicating an initial exponential growth phase" of the main text, the Gompertz model is the best-fit growth model overall.

References

[1] Laird AK. Dynamics of tumour growth. British journal of cancer. 1964;18(3):490.

[2] Gompertz B. On the nature of the function expressive of the law of human mortality, and on a new mode of determining the value of life contingencies. In a letter to Francis Baily, Esq. FRS &c. Philosophical transactions of the Royal Society of London. 1825;(115):513–583.

[3] Norton L, Simon R, Brereton HD, Bogden AE. Predicting the course of Gompertzian growth. Nature. 1976;264(5586):542–545.

[4] Norton L. A Gompertzian model of human breast cancer growth. Cancer research. 1988;48(24 Pt 1):7067–7071.

[5] Norton L. Conceptual and practical implications of breast tissue geometry: toward a more effective, less toxic therapy. The Oncologist. 2005;10(6):370–381.

[6] Marušić M, Bajzer Ž, Freyer J, Vuk-Pavlović S. Analysis of growth of multicellular tumour spheroids by mathematical models. Cell proliferation. 1994;27(2):73–94.

[7] Marušić M, Vuk-Pavlovic S, Freyer JP, et al. Tumor growth in vivo and as multicellular spheroids compared by mathematical models. Bulletin of Mathematical Biology. 1994;56(4):617–631.

[8] Olea N, Villalobos M, Nunez M, Elvira J, de Almodovar JR, Pedraza V. Evaluation of the growth rate of MCF-7 breast cancer multicellular spheroids using three mathematical models. Cell Proliferation. 1994;27(4):213–223.

[9] Benzekry S, Lamont C, Beheshti A, Tracz A, Ebos JM, Hlatky L, et al. Classical mathematical models for description and prediction of experimental tumor growth. PLoS Computational Biology. 2014;10(8):e1003800.

[10] Vaghi C, Rodallec A, Fanciullino R, Ciccolini J, Mochel JP, Mastri M, et al. Population modeling of tumor growth curves and the reduced Gompertz model improve prediction of the age of experimental tumors. PLoS Computational Biology. 2020;16(2):e1007178.

[11] Ghaffari Laleh N, Loeffler CML, Grajek J, Staňková K, Pearson AT, Muti HS, et al. Classical mathematical models for prediction of response to chemotherapy and immunotherapy. PLoS Computational Biology. 2022;18(2):e1009822.

[12] Simeoni M, Magni P, Cammia C, De Nicolao G, Croci V, Pesenti E, et al. Predictive pharmacokinetic-pharmacodynamic modeling of tumor growth kinetics in xenograft models after administration of anticancer agents. Cancer Research. 2004;64(3):1094–1101.

[13] Parra-Guillen ZP, Mangas-Sanjuan V, Garcia-Cremades M, Troconiz IF, Mo G, Pitou C, et al. Systematic modeling and design evaluation of unperturbed tumor dynamics in xenografts. Journal of Pharmacology and Experimental Therapeutics. 2018;366(1):96–104.

[14] Vaidya VG, Alexandro Jr FJ. Evaluation of some mathematical models for tumor growth. International Journal of Bio-medical Computing. 1982;13(1):19–35.

[15] Talkington A, Durrett R. Estimating tumor growth rates in vivo. Bulletin of Mathematical Biology. 2015;77:1934–1954.

[16] Mandonnet E, Delattre JY, Tanguy ML, Swanson KR, Carpentier AF, Duffau H, et al. Continuous growth of mean tumor diameter in a subset of grade II gliomas. Annals of Neurology: Official Journal of the American Neurological Association and the Child Neurology Society. 2003;53(4):524–528.

[17] West GB, Brown JH, Enquist BJ. A general model for ontogenetic growth. Nature. 2001;413(6856):628–631.

[18] Sarapata EA, De Pillis L. A comparison and catalog of intrinsic tumor growth models. Bulletin of Mathematical Biology. 2014;76:2010–2024.

[19] Rodriguez-Brenes IA, Komarova NL, Wodarz D. Tumor growth dynamics: insights into evolutionary processes. Trends in Ecology & Evolution. 2013;28(10):597–604.

[20] Gerlee P. The model muddle: in search of tumor growth laws. Cancer Research. 2013;73(8):2407–2411.

Category	Description
A	No merging or splitting issue
B	Slight merging or splitting issue
C	Boundary case
X	Significant merging issue
Y	Significant splitting or partial identification issue
Z	Other issue

Table 1: Labels used to categorize tracked organoids depending on whether they had been merged with a nearby organoid by the automated NN image analysis or they had been split into two or more organoids by the NN (which was assessed visually).

	Accepted by the filter					
	A	B	C	X	Y	Z
UK well 1	22	10	0	1	0	0
UK well 2	12	7	1	1	0	3
UK well 3	12	10	2	0	0	0
UK well 4	6	6	0	1	1	0
UP well 1	34	9	2	3	1	0
UP well 2	17	6	1	0	1	1
UP well 3	21	10	0	1	1	0
UP well 4	16	12	1	1	2	0
US well 1	7	9	0	1	1	0
US well 2	11	5	0	0	2	1
US well 3	5	9	4	0	1	1
US well 4	2	12	2	0	0	0
	Rejected by the filter					
	A	B	C	X	Y	Z
UK well 1	5	2	0	5	3	4
UK well 2	0	0	2	12	3	2
UK well 3	0	2	2	3	2	0
UK well 4	1	1	2	2	6	0
UP well 1	20	18	10	19	22	0
UP well 2	1	0	1	17	7	1
UP well 3	4	5	8	12	5	3
UP well 4	10	1	6	36	17	6
US well 1	1	4	0	10	8	3
US well 2	1	1	0	0	13	0
US well 3	1	1	2	7	10	4
US well 4	0	3	2	5	9	1

Table 2: Results of organoid labeling according to a manual review of four experimental wells for each patient organoid. The upper half of the table shows the number of organoids assigned to each label for organoids accepted by the data filter. The lower half shows the same numbers for organoids rejected by the data filter.

	Precision (C acceptable)	Precision (C unacceptable)
UK well 1	97,0%	97,0%
UK well 2	83,3%	79,2%
UK well 3	93,9%	93,9%
UK well 4	100,0%	91,7%
UP well 1	91,8%	87,8%
UP well 2	92,3%	88,5%
UP well 3	93,5%	90,3%
UP well 4	85,7%	85,7%
US well 1	88,9%	88,9%
US well 2	84,2%	84,2%
US well 3	90,0%	70,0%
US well 4	100,0%	87,5%
Average	91,7%	87,1%

Table 3: Precision of the data filter constructed in Section 2, which is the percentage of organoids accepted by the filter with slight or no merging or splitting errors. The precision is shown both considering the boundary case C as acceptable (i.e. A, B and C are considered acceptable) and as unacceptable (i.e. A and B are considered acceptable).

	vB 0.8	vB 0.85	vB 0.9	vB 0.95	vB 0.99	Gomp
UK–1	0.0725	0.0543	0.0431	0.0362	0.0319	0.0309
UK–2	0.0751	0.0544	0.0397	0.0303	0.0249	0.0237
UP–1	0.0517	0.0436	0.0378	0.0342	0.0334	0.0317
UP–2	0.0343	0.0270	0.0227	0.0208	0.0204	0.0201
US–1	0.0606	0.0512	0.0431	0.0369	0.0343	0.0334
US–2	0.1179	0.1115	0.1067	0.1036	0.1016	0.1011
US–3	0.0675	0.0598	0.0544	0.0510	0.0505	0.0490

Table 4: Mean normalized fitting error for the von Bertalanffy (vB) model with exponents $\gamma \in \{0.8, 0.85, 0.9, 0.95, 0.99\}$ as well as the Gompertz model across individual organoids in the UK/UP/US datasets. Note that as γ approaches 1, the fitting error for the von Bertalanffy model decreases and approaches the fitting error for the Gompertz model.

	vB 0.8	vB 0.85	vB 0.9	vB 0.95	vB 0.99
UK–1	0.0360	0.0229	0.0131	0.0060	0.0011
UK–2	0.0505	0.0333	0.0194	0.0087	0.0016
UP–1	0.0215	0.0150	0.0091	0.0042	0.0027
UP–2	0.0156	0.0102	0.0060	0.0028	0.0008
US–1	0.0237	0.0165	0.0101	0.0043	0.0010
US–2	0.0164	0.0117	0.0074	0.0035	0.0007
US–3	0.0174	0.0118	0.007	0.0032	0.0023

Table 5: Average difference between the fitted growth curves for the von Bertalanffy models with $\gamma \in \{0.8, 0.85, 0.9, 0.95, 0.99\}$ on the one hand and the Gompertz model on the other hand across individual organoids in the UK/UP/US datasets. For each organoid and each von Bertalanffy model, the difference is computed as $\int_0^5 |N_{\text{Gomp}}(\tau_{\text{Gomp}} + t) - N_{\text{vB}}(\tau_{\text{vB}} + t)|dt / \int_0^5 N_{\text{Gomp}}(\tau_{\text{Gomp}} + t)dt$, where $t \mapsto N_{\text{Gomp}}(t)$ is the fitted Gompertz curve and $t \mapsto N_{\text{vB}}(t)$ is the fitted von Bertalanffy curve.

	Exp	PL 1/2	PL 2/3	PL 3/4	Gomp	Log	vB 1/2	vB 2/3	vB 3/4
UK–1	13.45	20.78	15.93	13.53	-20.76	**-20.93**	16.65	7.72	0.34
UK–2	13.36	22.56	18.03	15.21	-22.11	**-22.85**	18.63	9.59	2.01
UP–1	21.59	19.46	17.65	17.04	**-15.95**	-9.47	6.43	-1.77	-6.45
UP–2	23.88	22.08	17.77	17.37	**-16.31**	-10.96	15.85	3.76	-2.92
US–1	13.32	13.30	10.40	10.91	**-15.65**	-13.26	4.89	-6.4	-9.75
US–2	15.28	12.39	12.45	12.32	**-8.15**	-5.27	-2.30	-3.63	-5.03
US–3	15.91	12.96	13.40	13.02	**-14.22**	-12.72	-3.59	-7.97	-11.29

Table 6: Average BIC obtained by fitting each mathematical model to each individual organoid in the UK/UP/US datasets, under alternative volume conversion method (i) in Section 4. The best-fit model for each dataset is indicated by bold.

	Exp	PL 1/2	PL 2/3	PL 3/4	Gomp	Log	vB 1/2	vB 2/3	vB 3/4
UK–1	11.92	19.09	14.05	11.64	-20.71	**-21.19**	14.93	5.79	-3.14
UK–2	12.72	21.67	16.64	13.62	**-19.90**	-19.65	18.56	10.40	2.82
UP–1	20.36	18.03	16.29	15.95	**-17.61**	-12.30	5.20	-4.56	-9.55
UP–2	23.13	19.81	16.60	16.02	**-20.58**	-14.54	12.19	-1.61	-7.22
US–1	10.93	11.26	10.11	9.37	**-18.90**	-18.52	-1.35	-6.29	-9.72
US–2	13.96	13.33	12.86	13.15	**-7.19**	-5.07	3.10	-0.80	-1.40
US–3	13.89	13.73	13.40	12.65	**-14.56**	-12.82	-3.35	-5.90	-7.98

Table 7: Average BIC obtained by fitting each mathematical model to each individual organoid in the UK/UP/US datasets, under alternative volume conversion method (ii) in Section 4. The best-fit model for each dataset is indicated by bold.

	Exp	PL 1/2	PL 2/3	PL 3/4	Gomp	Log	vB 1/2	vB 2/3	vB 3/4
UK–1	-1.84	-0.09	-2.96	-5.07	**-24.69**	-24.44	-2.45	-7.06	-12.29
UK–2	-1.76	0.36	-1.91	-3.29	**-24.78**	-24.60	-1.06	-5.94	-10.77
UP–1	1.75	-3.53	-4.12	-3.54	**-22.68**	-18.58	-10.54	-15.16	-17.72
UP–2	1.75	-3.27	-5.79	-5.11	**-24.53**	-20.29	-6.60	-13.6	-17.01
US–1	-0.08	-4.41	-3.05	-3.42	**-22.77**	-20.93	-11.15	-15.38	-17.39
US–2	1.43	-2.66	-2.35	-1.92	**-15.86**	-14.19	-9.47	-12.32	-13.40
US–3	0.27	-2.72	-3.36	-2.85	**-21.46**	-19.56	-13.58	-16.54	-18.27

Table 8: Average BIC obtained by fitting each mathematical model to each individual organoid in the UK/UP/US datasets, under a logarithmic transformation of the models and the data (Section 5). The best-fit model for each dataset is indicated by bold.

	Gompertz	Logistic	vB 1/2	vB 2/3	vB 3/4
UK–1	**0.0071**	0.0072	0.0156	0.0123	0.0106
		1.0118	*2.1874*	*1.7277*	*1.4897*
UK–2	**0.0047**	0.0047	0.0128	0.0098	0.0082
		1.005	*2.7052*	*2.0742*	*1.7454*
UP–1	**0.0035**	0.0042	0.0065	0.0053	0.0047
		1.1978	*1.8519*	*1.4961*	*1.3311*
UP–2	**0.0015**	0.0022	0.0037	0.0026	0.0022
		1.4223	*2.4176*	*1.713*	*1.4167*
US–1	**0.0058**	0.0065	0.0112	0.0089	0.0078
		1.1135	*1.9166*	*1.5245*	*1.3459*
US–2	**0.0167**	0.0181	0.0225	0.0202	0.0191
		1.0872	*1.3479*	*1.2123*	*1.1436*
US–3	**0.0057**	0.0063	0.01	0.0082	0.0075
		1.1071	*1.7615*	*1.4448*	*1.3159*

Table 9: Mean normalized fitting error for the Gompertz, logistic and von Bertalanffy (vB) models with exponents $\gamma \in \{1/2, 2/3, 3/4\}$ across individual organoids in the UK/UP/US datasets, under a logarithmic transformation of the models and the data (Section 5). The best-fit model for each dataset is indicated by bold. The number in italics shows the mean normalized error relative to the Gompertz model error.
