# Supplementary figures and images for: Understanding patient-derived tumor organoid growth through an integrated imaging and mathematical modeling framework
Source: PLoS Comput Biol. 2024 Aug 2;20(8):e1012256. doi: 10.1371/journal.pcbi.1012256 (PMC11324155; doi:10.1371/journal.pcbi.1012256)

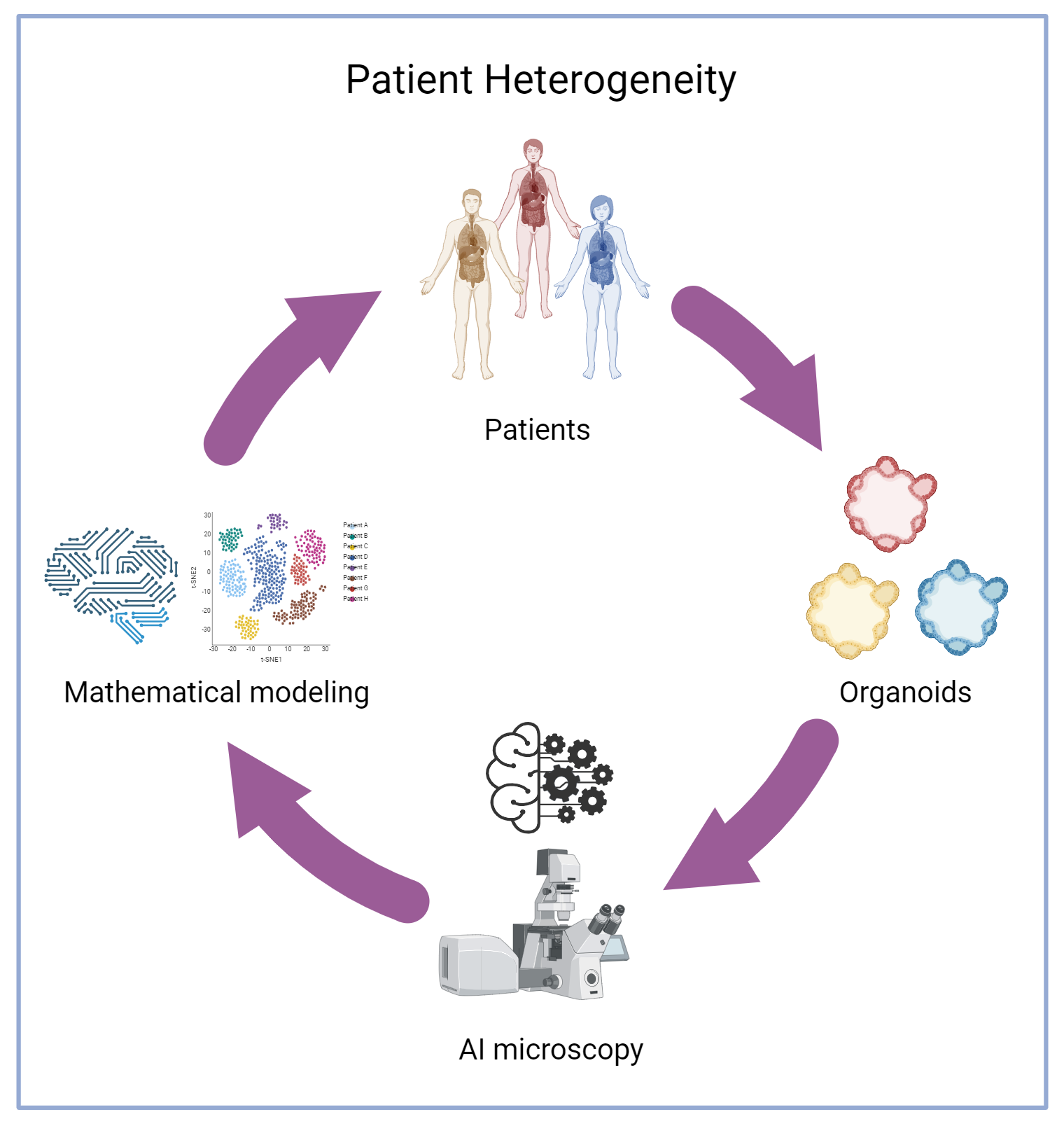

Supplement: S1 Fig — Image was generated using the BioRender software. (TIF) [file pcbi.1012256.s002.tif]

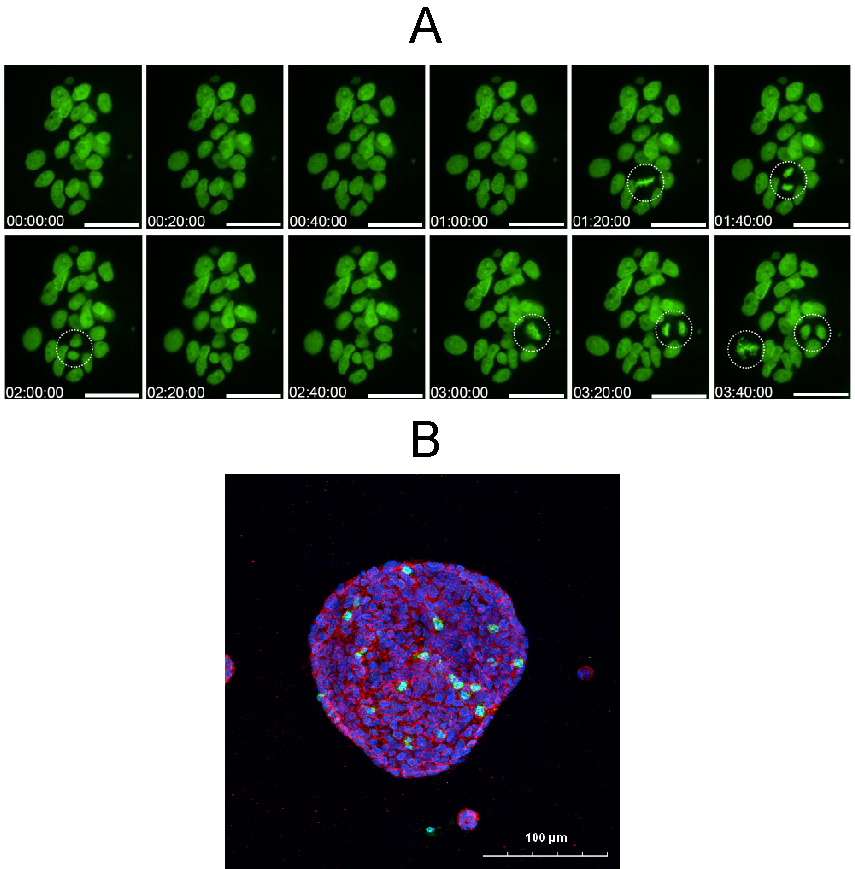

Supplement: S2 Fig — A: Live cell imaging of US-GFP organoid. Cell division events are highlighted. Single Z plane at the center of organoid was imaged over time (every 20 minutes for 12 hours). Time stamp, hours: minutes: seconds. Scale bar, 20 micrometer. (S4 Video) B: Immunostaining of US organoid shows the actively proliferating cells (Green-labeled). Ki67 (Green): Cell proliferation, E-cadherin (Red): Cell junction, DAPI (Blue): Cell nuclei. (TIF) [file pcbi.1012256.s003.tif]

(a)

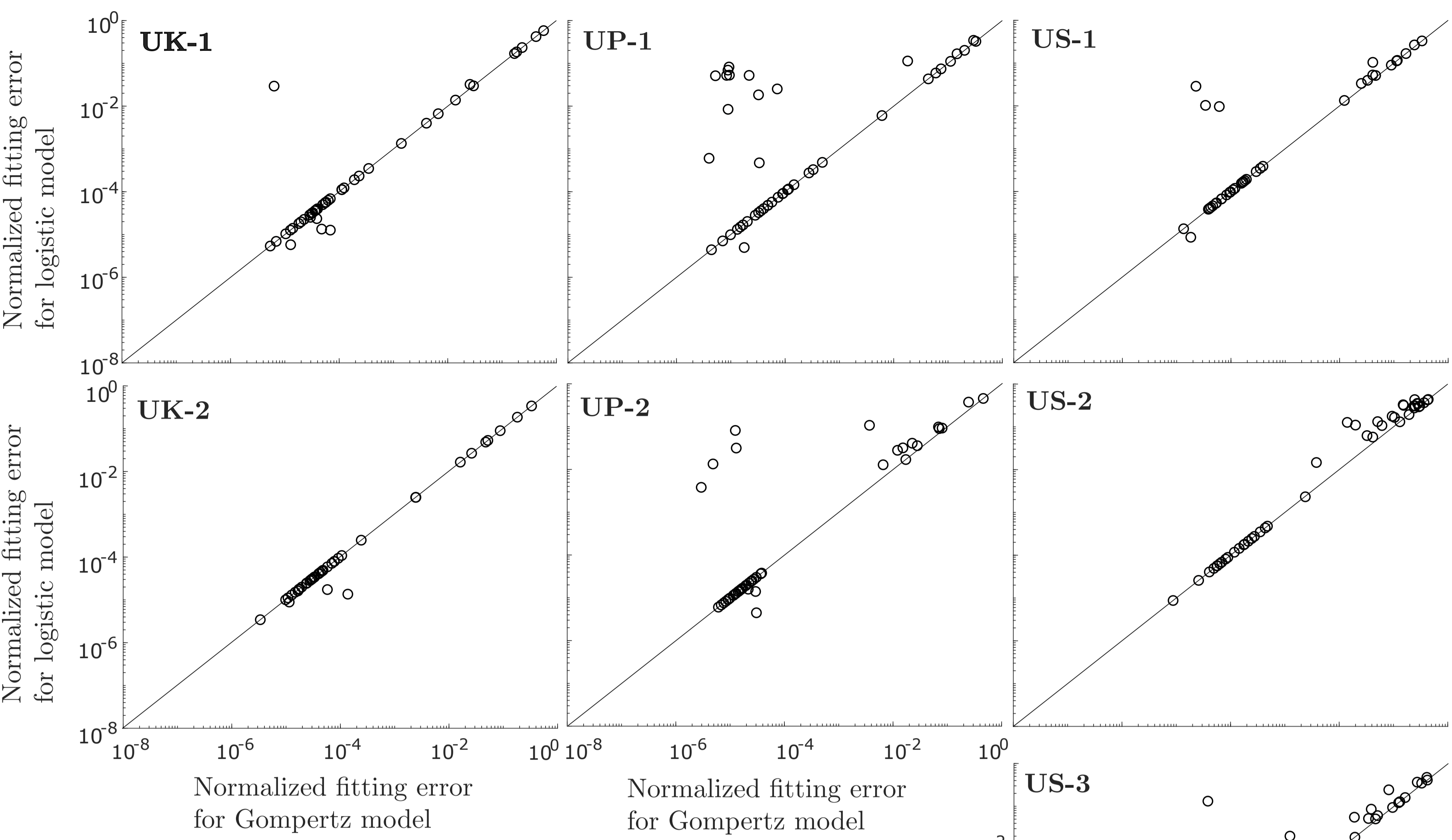

(b)

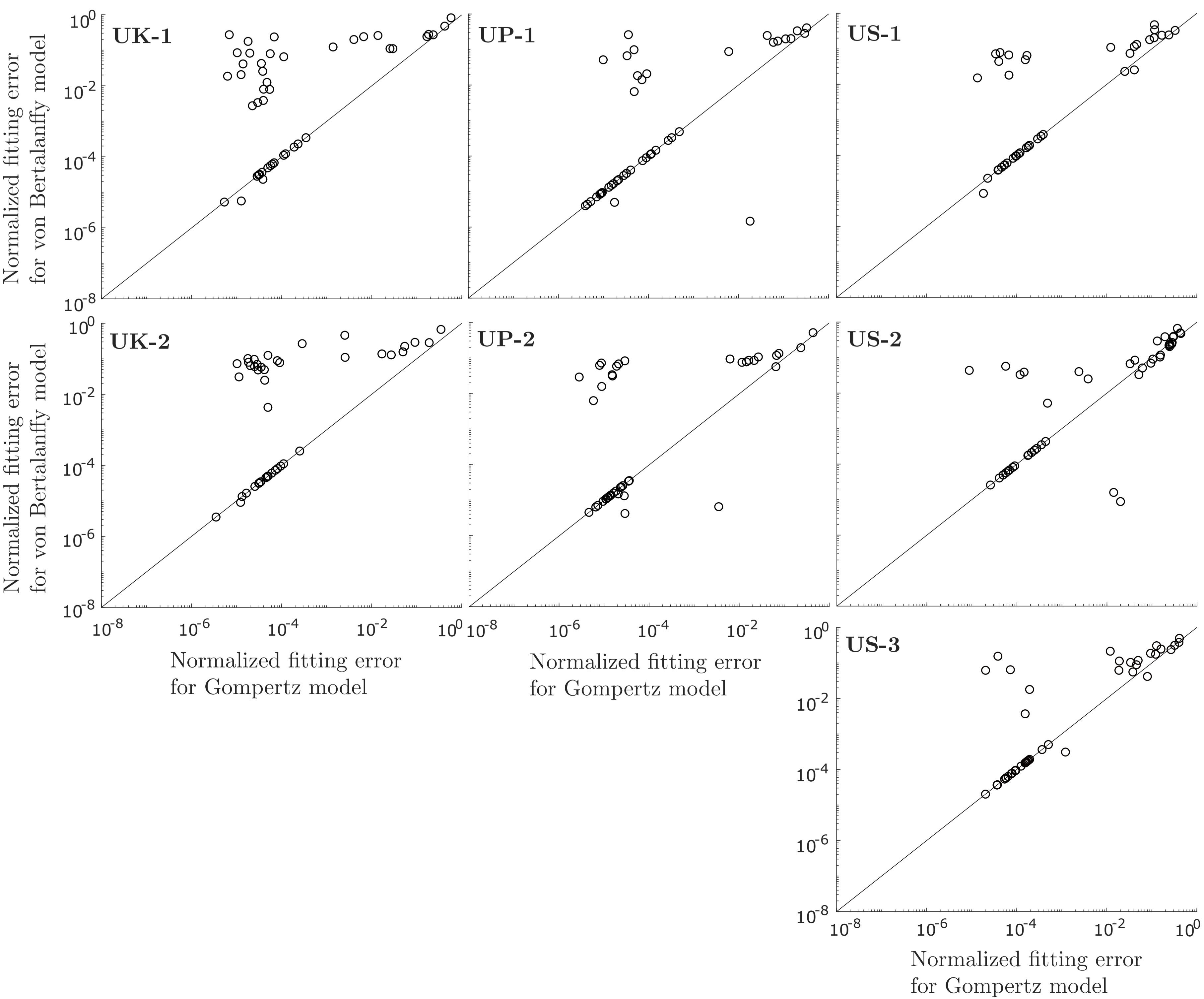

Supplement: S3 Fig — A: Comparison of normalized fitting errors (Section “Model selection”) individual organoids in the UK/UP/US datasets between the Gompertz and logistic models. Each dot represents a single organoid. B: Comparison of normalized fitting errors between the Gompertz model and the von Bertalanffy model with γ = 3/4. (PDF) [file pcbi.1012256.s004.pdf]

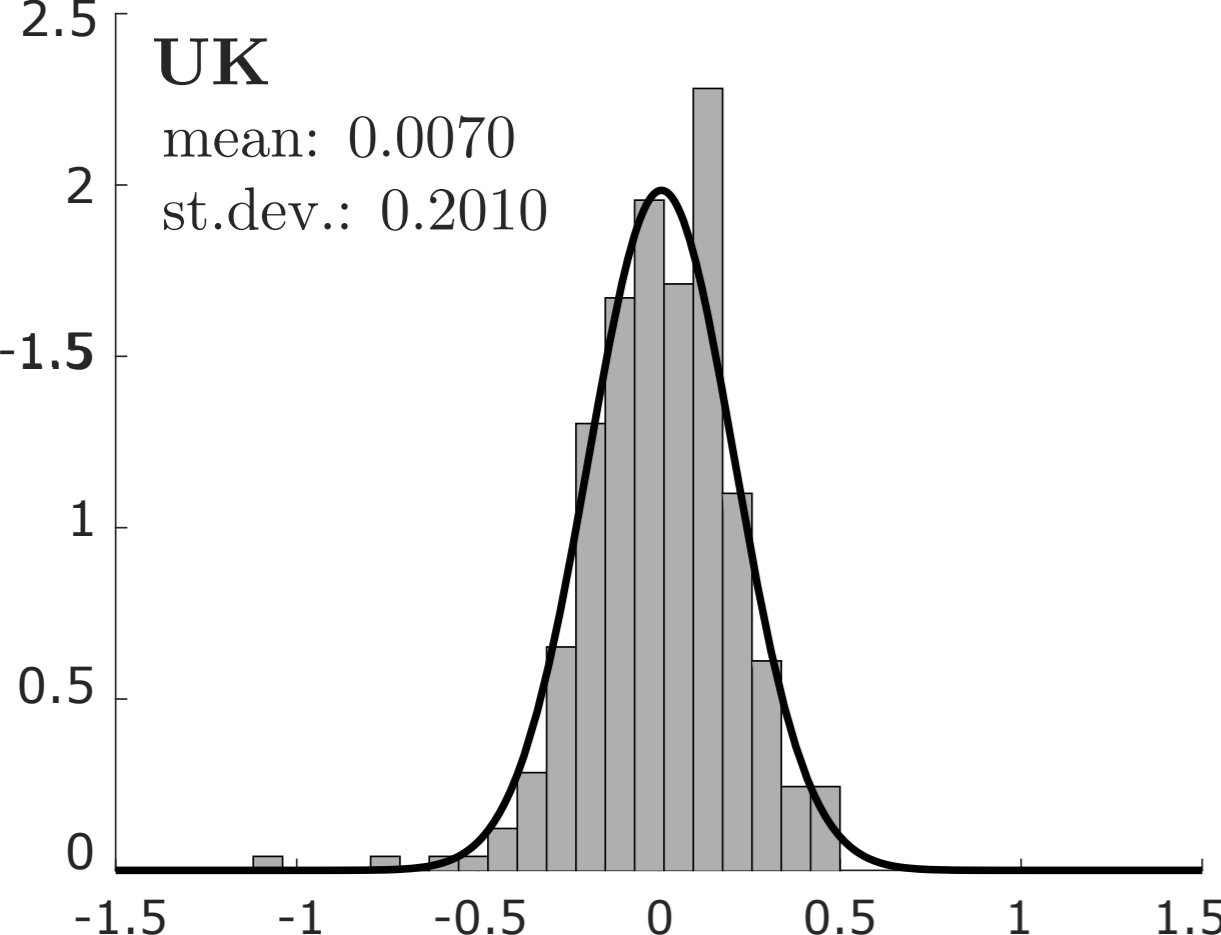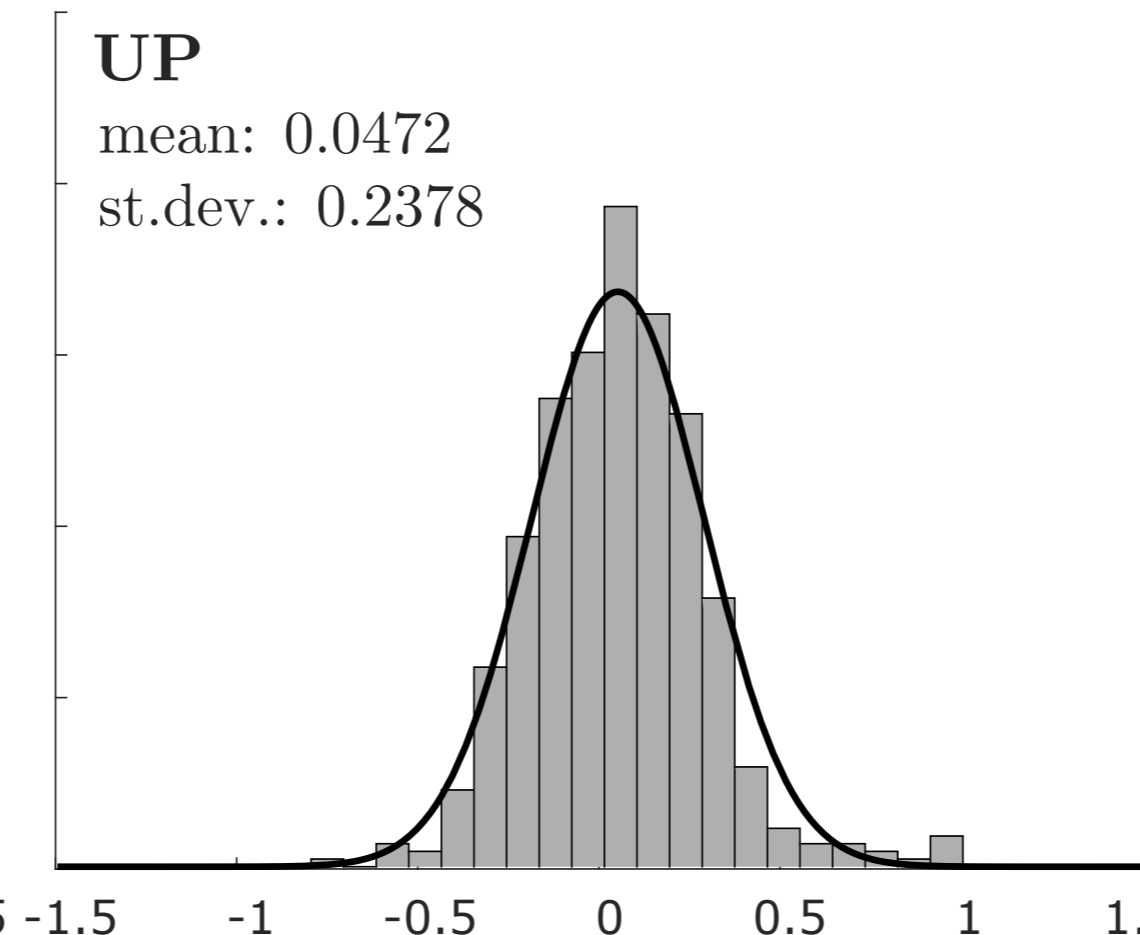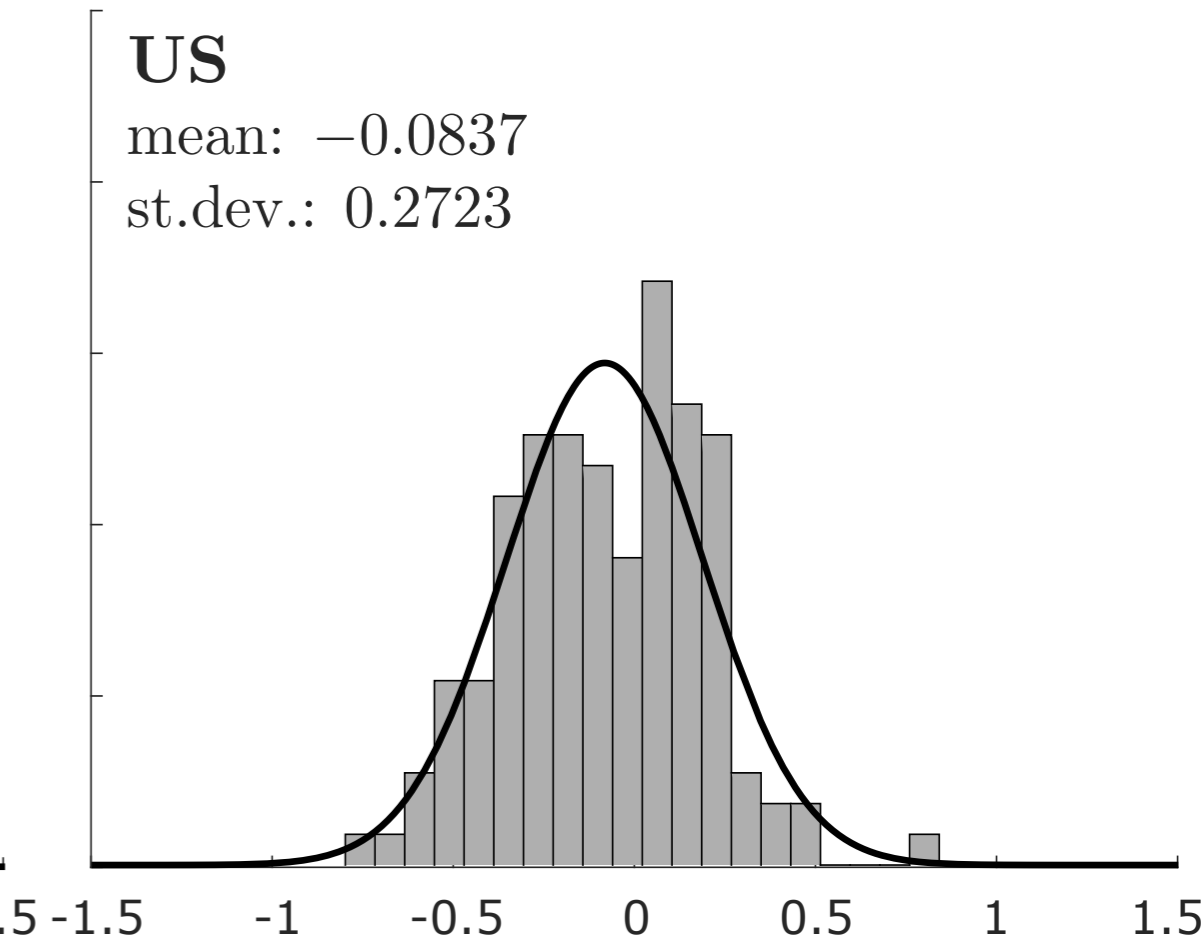

Supplement: S4 Fig — The different datasets for each patient have been combined. The mean and standard deviation of each distribution are shown. For each patient, the distribution of log10(a) is consistent with a normal distribution according to a Kolmogorov-Smirnov test at the 5% significance level, meaning that we fail to reject the null hypopthesis of normality. (PDF) [file pcbi.1012256.s005.pdf]

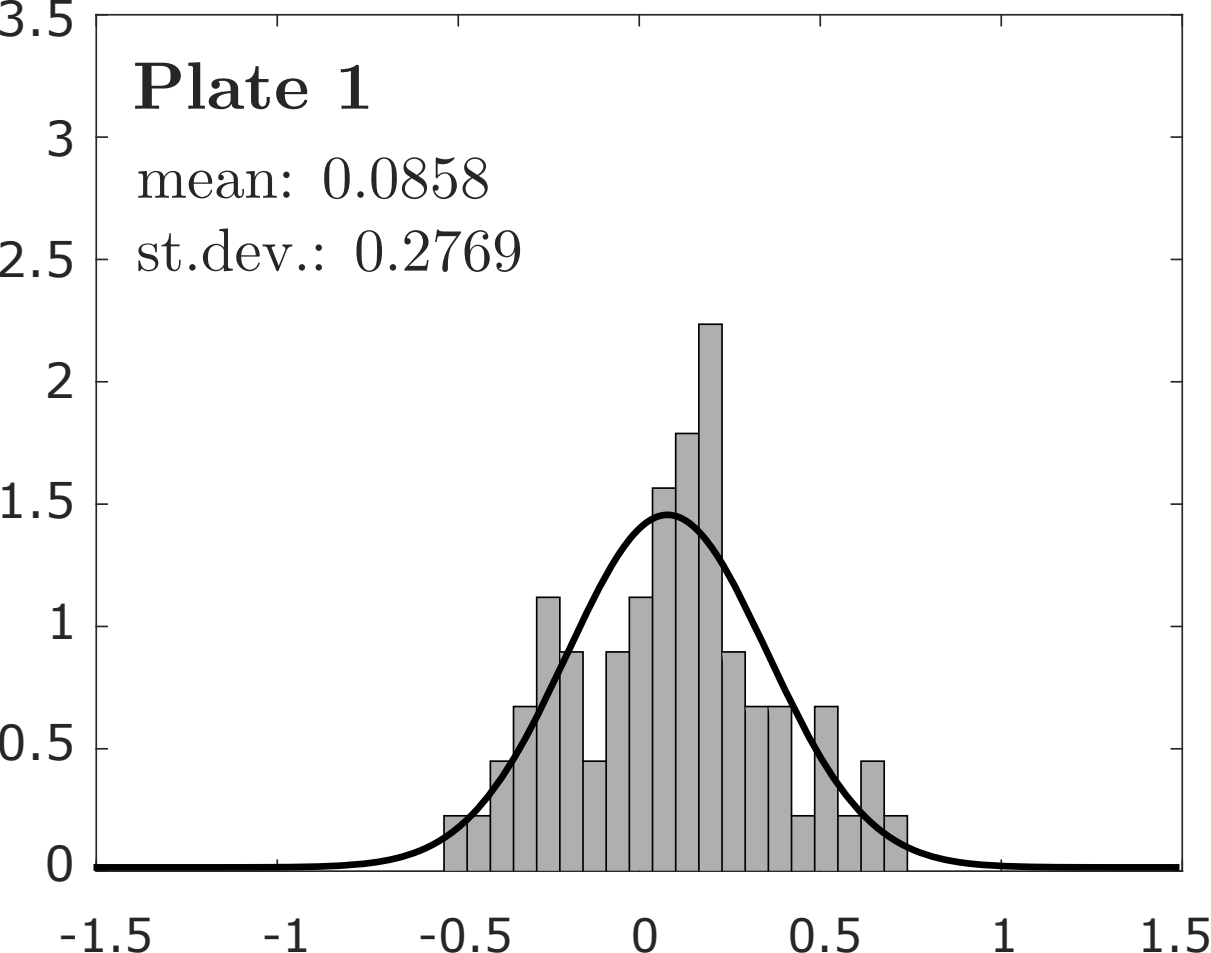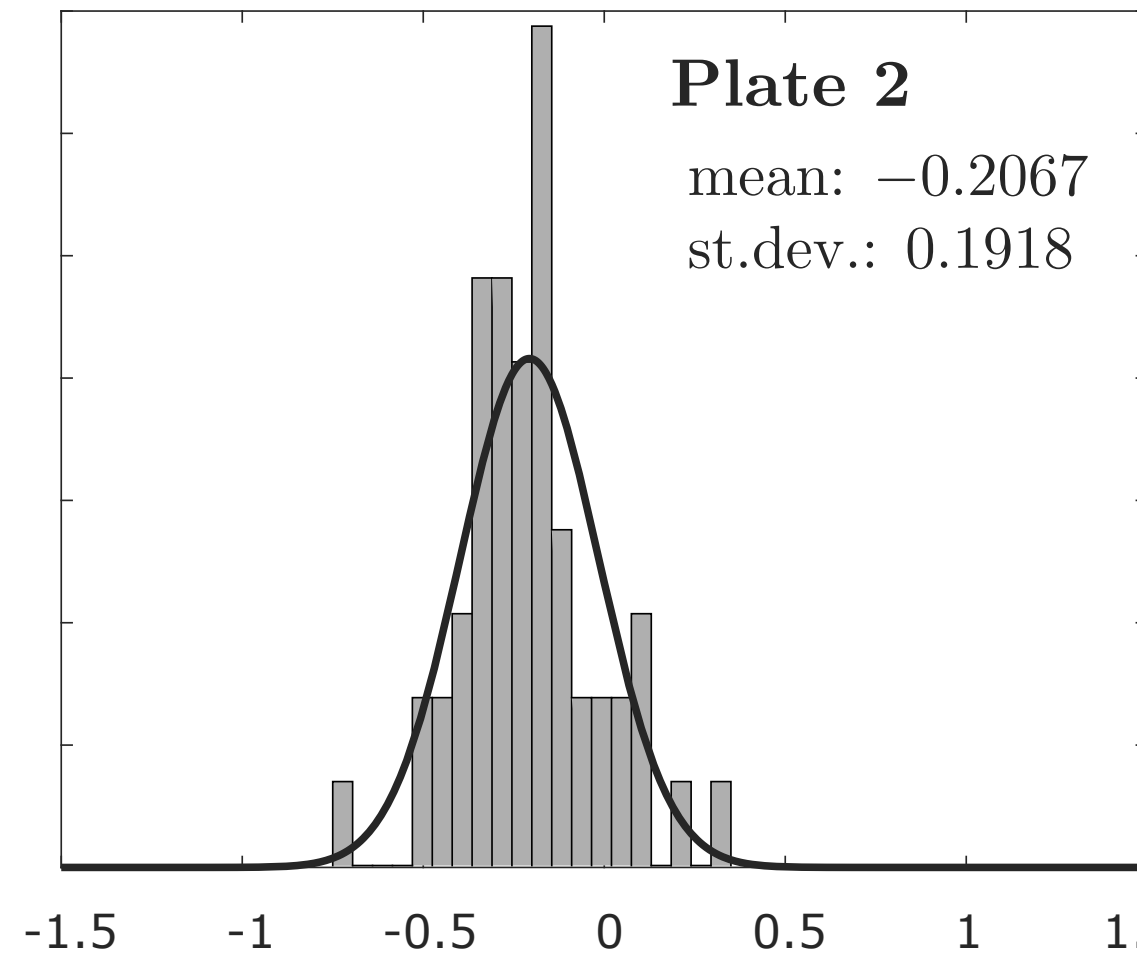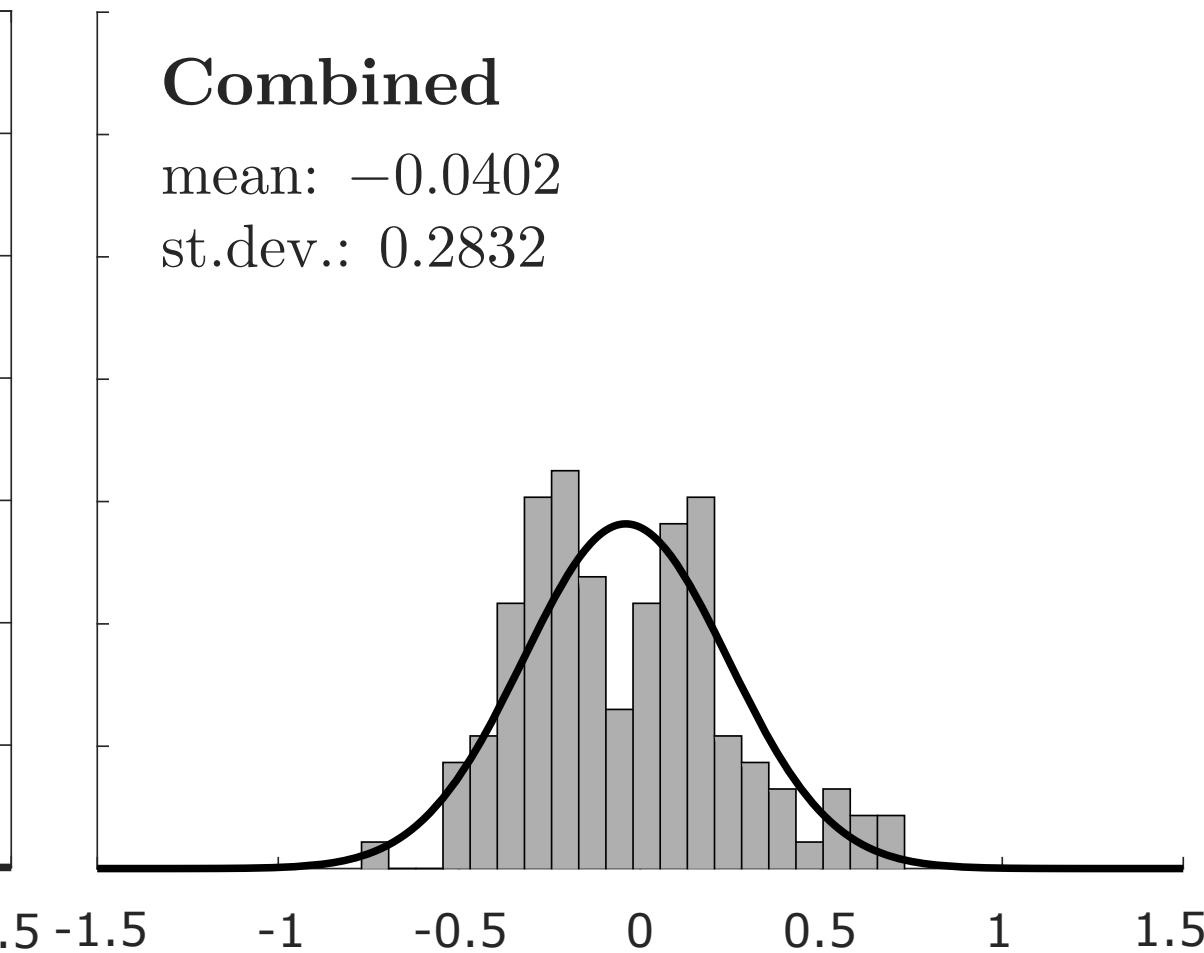

Supplement: S5 Fig — The mean and standard deviation of each distribution are shown. For each plate individually and for the two plates combined, the distribution of log10(a) is consistent with a normal distribution according to a Kolmogorov-Smirnov test at the 5% significance level. (PDF) [file pcbi.1012256.s006.pdf]

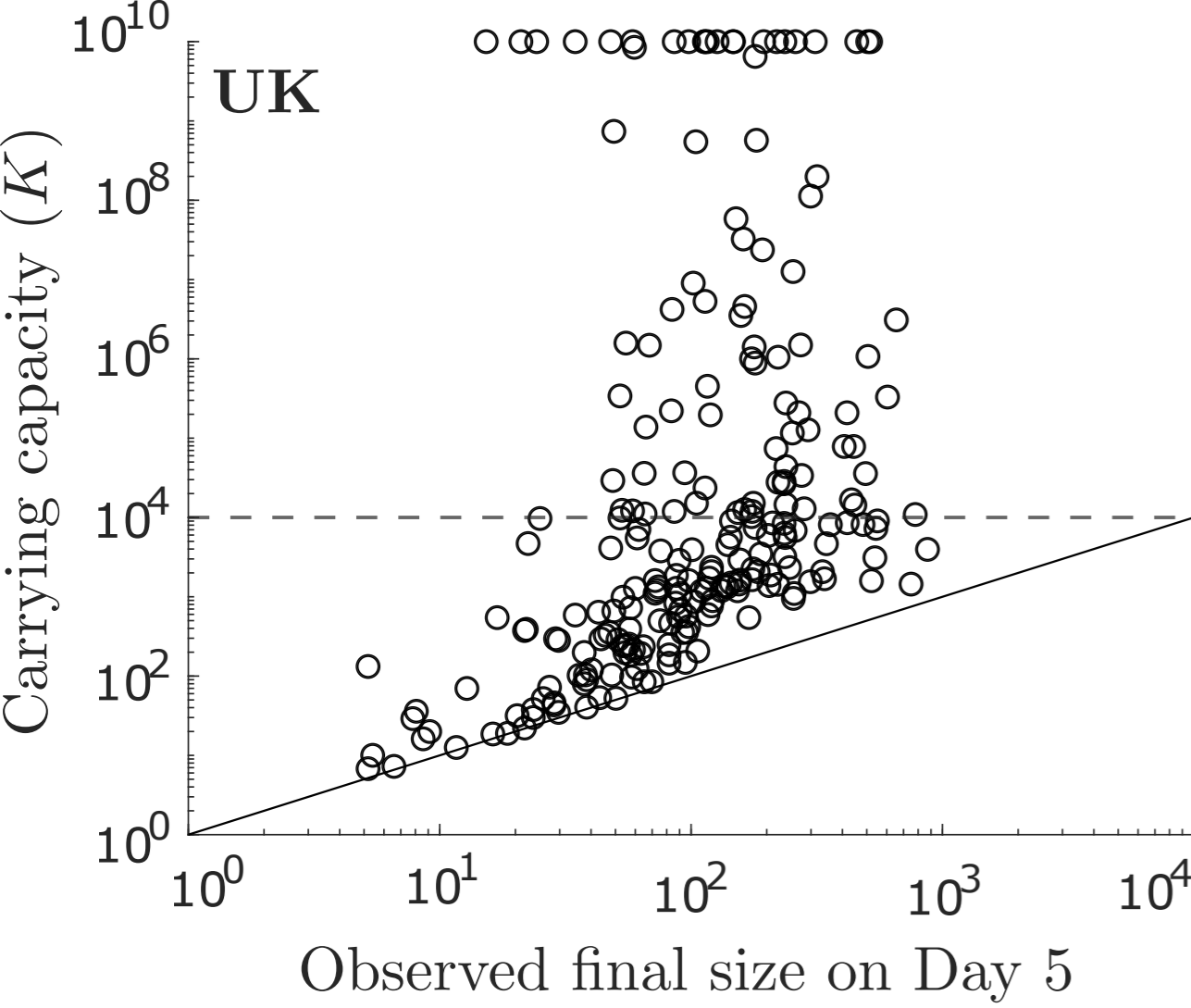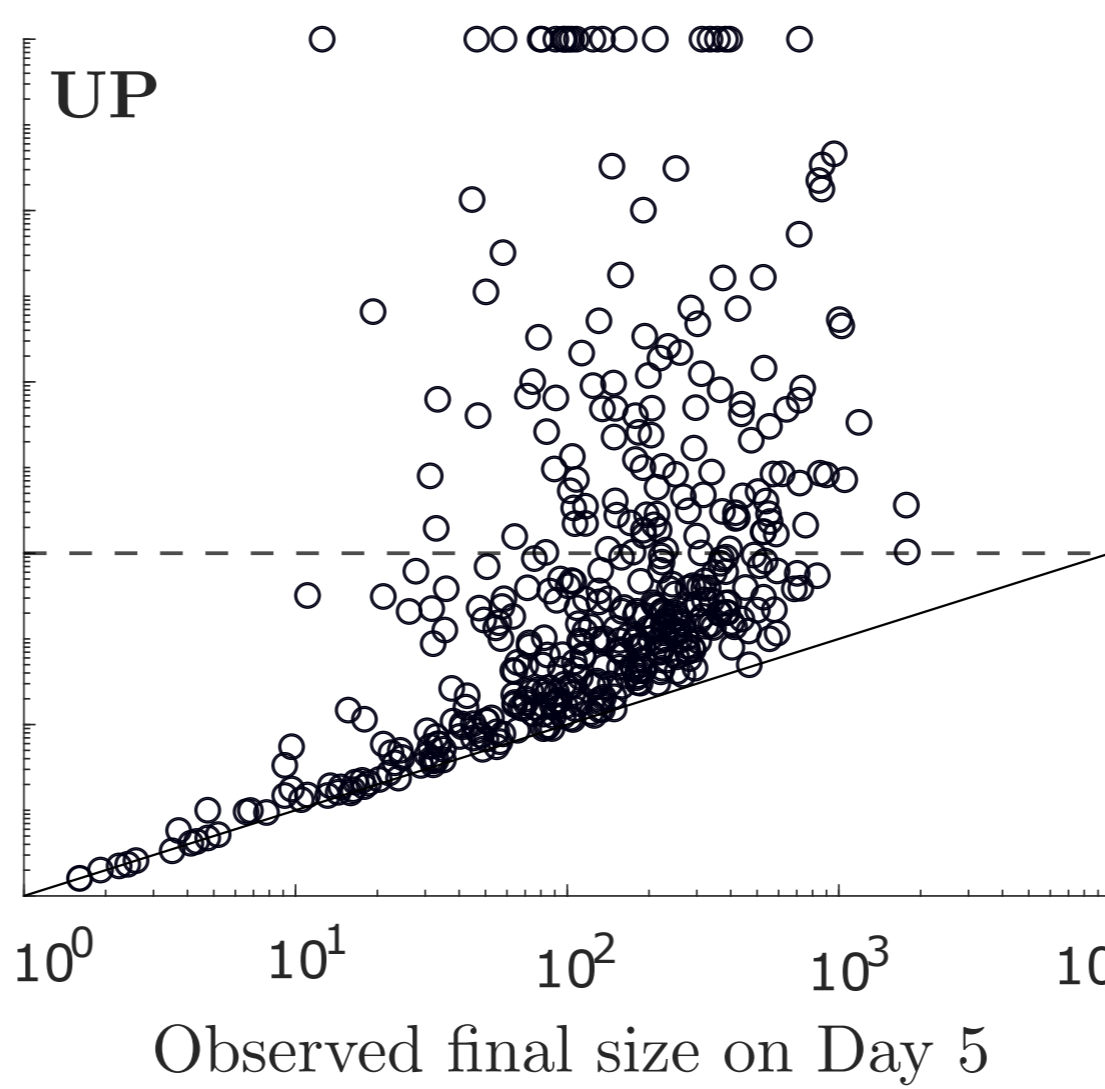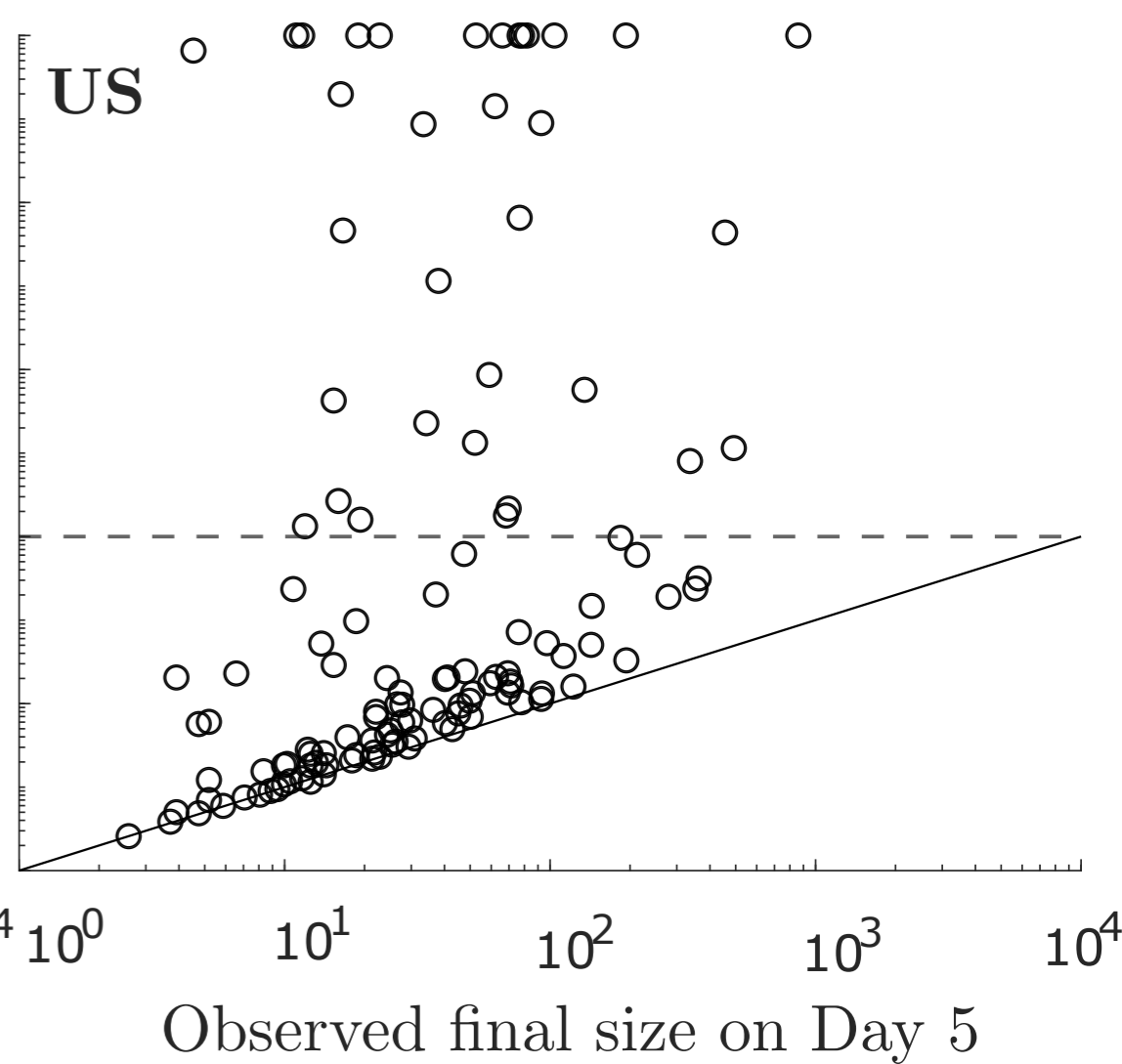

Supplement: S6 Fig — The datasets for each patient have been combined. Each dot represents a single organoid and only nonexponential organoids are considered (b > 10−4). Carrying capacities above 1010 cells are set to 1010. (PDF) [file pcbi.1012256.s007.pdf]

(a)

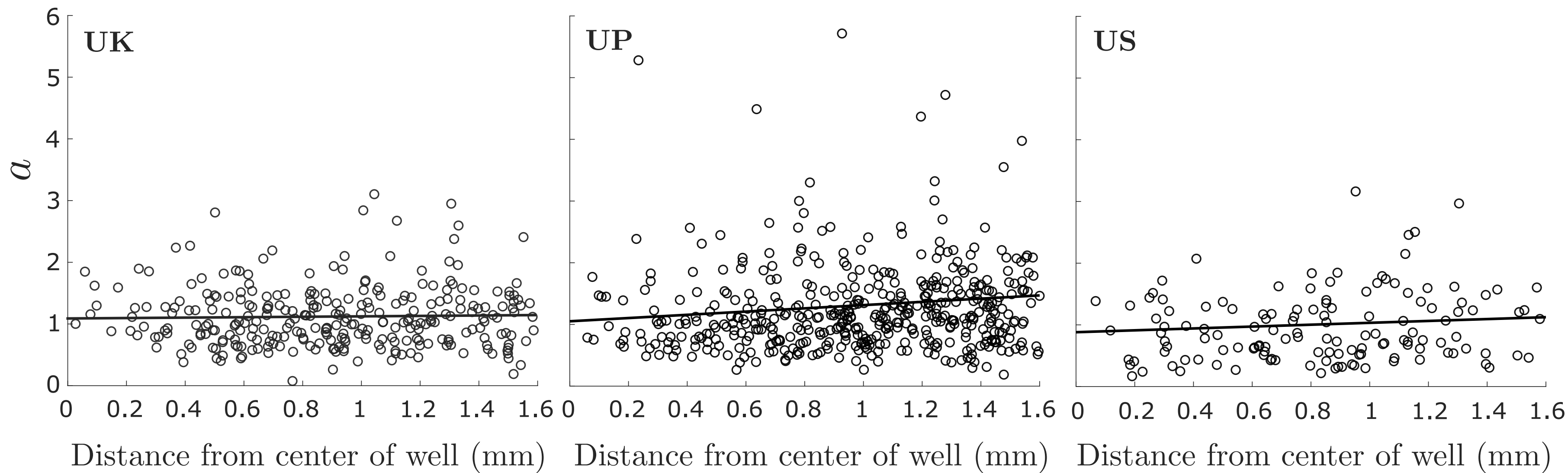

(b)

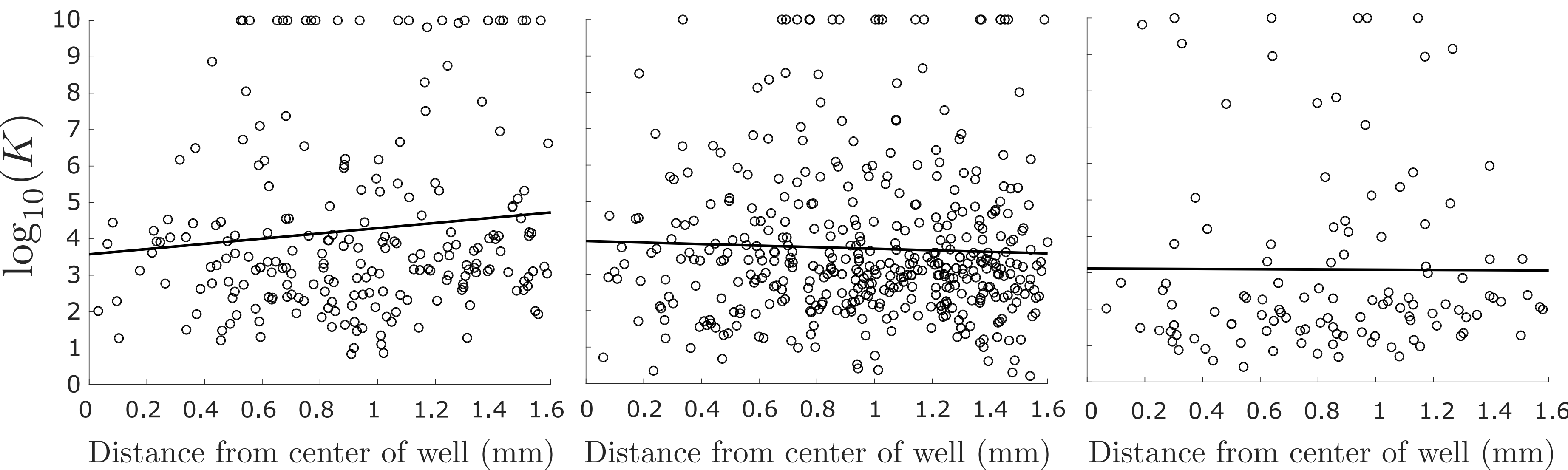

Supplement: S7 Fig — A: Comparison between the distance r of an organoid from the center of the experimental well and the initial exponential growth rate a of the organoid. Each dot represents a single organoid. The datasets for each patient have been combined. For each patient, a best-fit line is shown. The slope of the line cannot be distinguished from zero at the 5% significance level for any of the patient samples when a Bonferroni correction is applied. B: Comparison between the distance r of an organoid from the center of the experimental well and its estimated carrying capacity K according to the Gompertz model. Only nonexponential organoids are considered (b > 10−4), and carrying capacities above 1010 cells are set to 1010. For each patient, a best-fit line is shown. The slope of the line cannot be distinguished from zero at the 5% significance level for any of the patient samples, whether or not a Bonferroni correction is applied. (PDF) [file pcbi.1012256.s008.pdf]

(a)

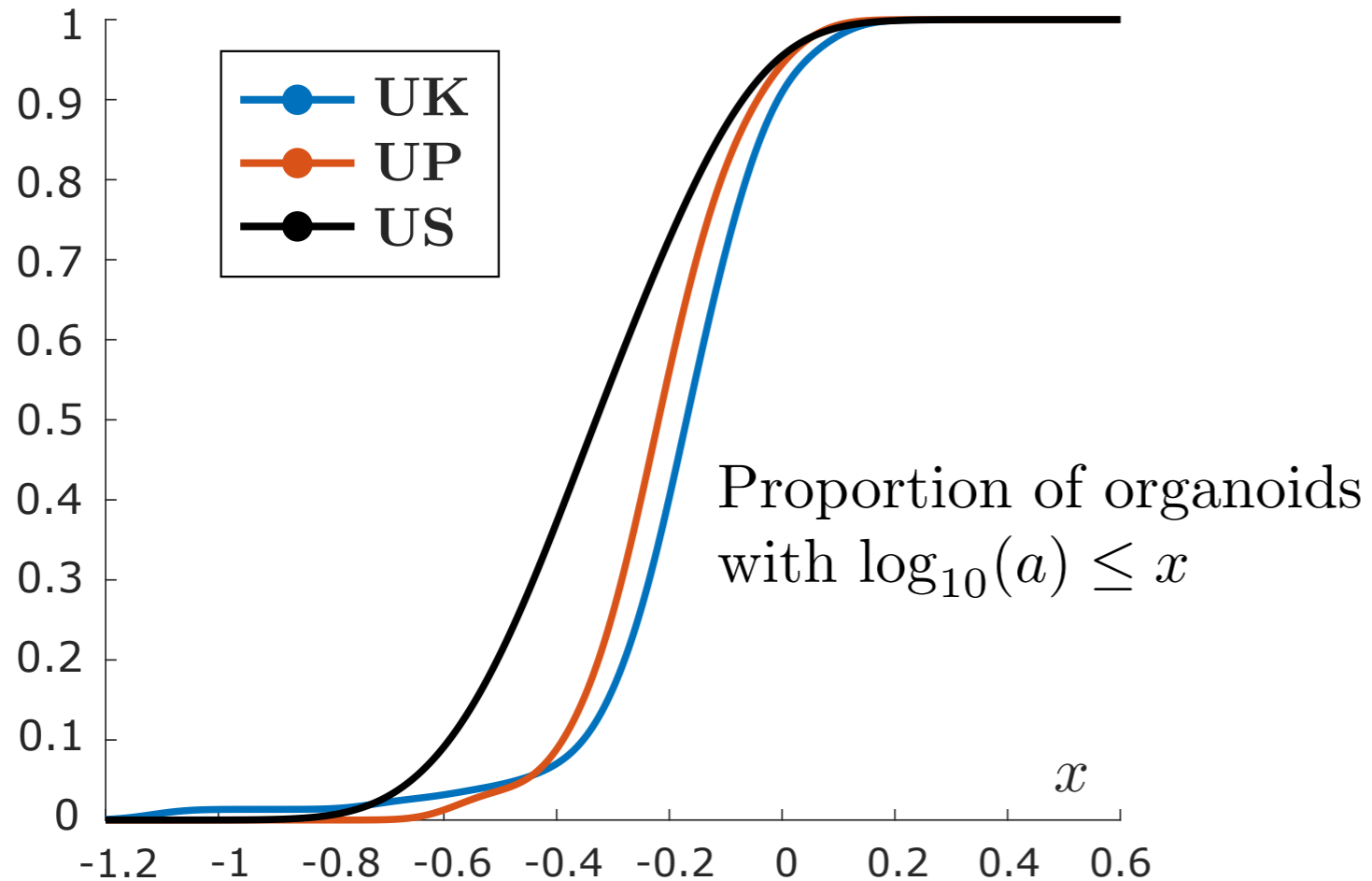

(b)

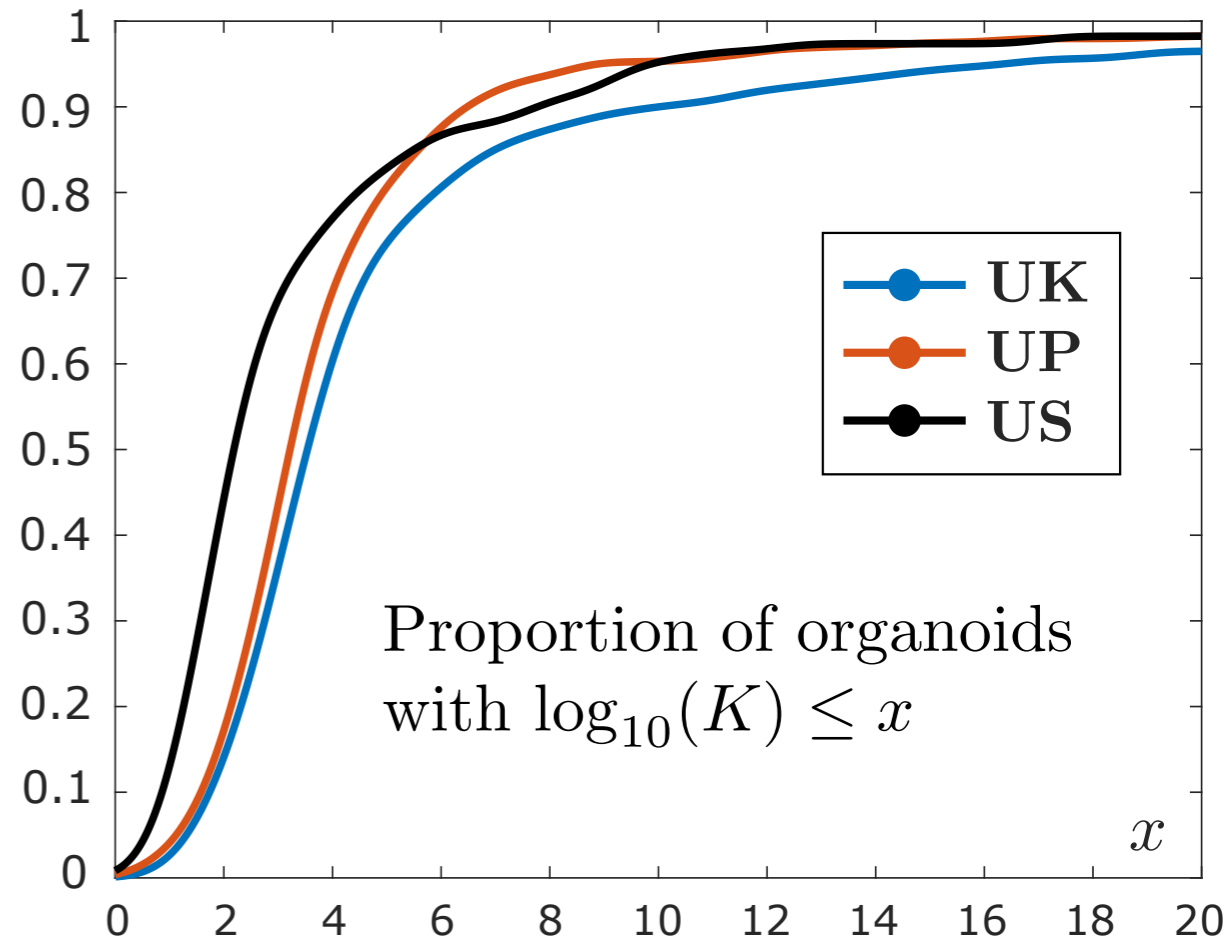

Supplement: S8 Fig — A: Comparison of the distributions of log10(a) between the different patient samples, where only exponential organoids are considered (b = 10−4), and the datasets for each patient have been combined. For each patient sample, the graph of the cumulative distribution function (CDF) of log10(a) is shown, which gives for each value of x the proportion of organoids satisfying log10(a) ≤ x. B: Comparison of the distributions of carrying capacities between the different patient samples, where only nonexponential organoids are considered (b > 10−4). For each patient sample, the graph of the CDF of log10(K) is shown. (PDF) [file pcbi.1012256.s009.pdf]

Proportion of organoids

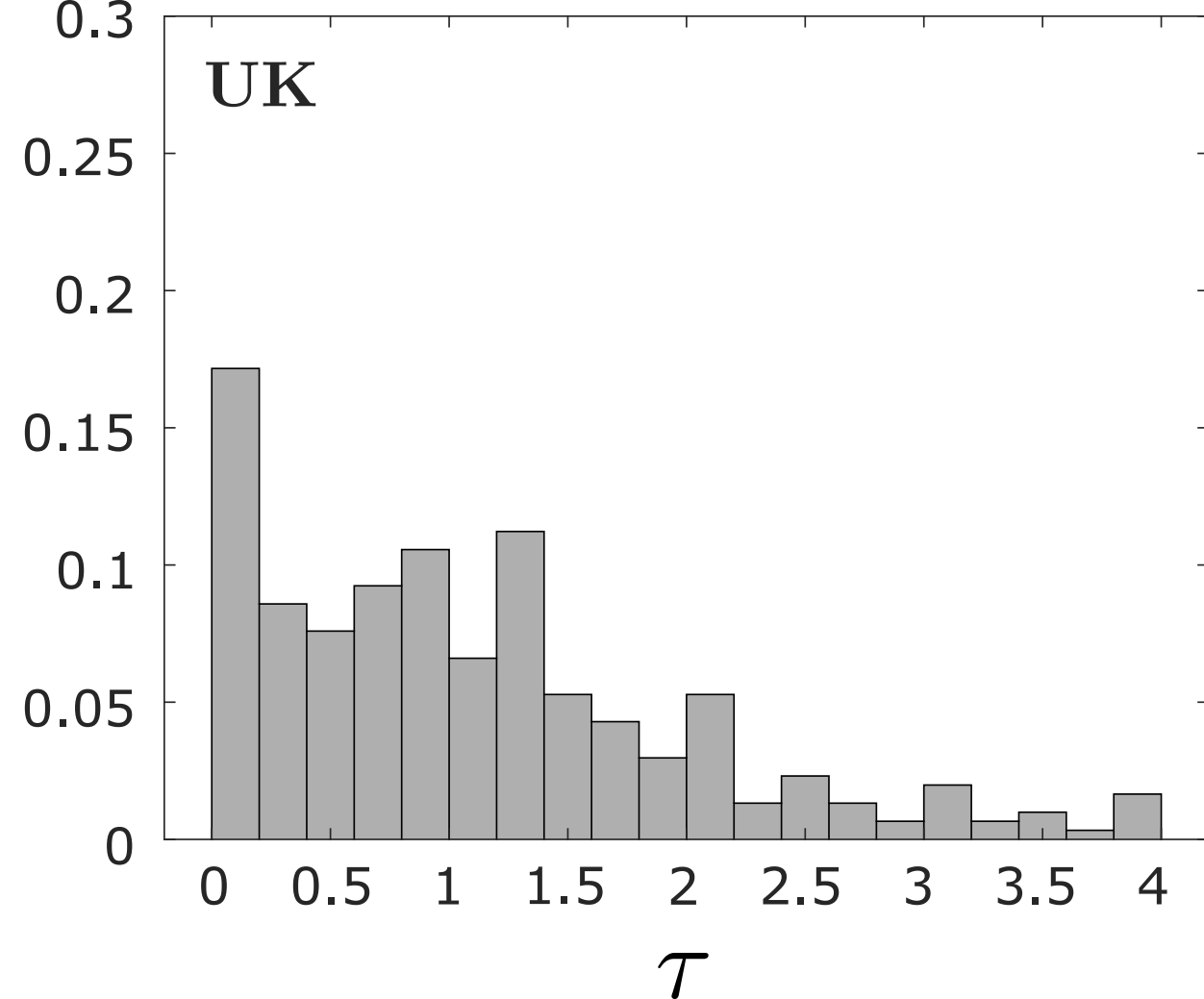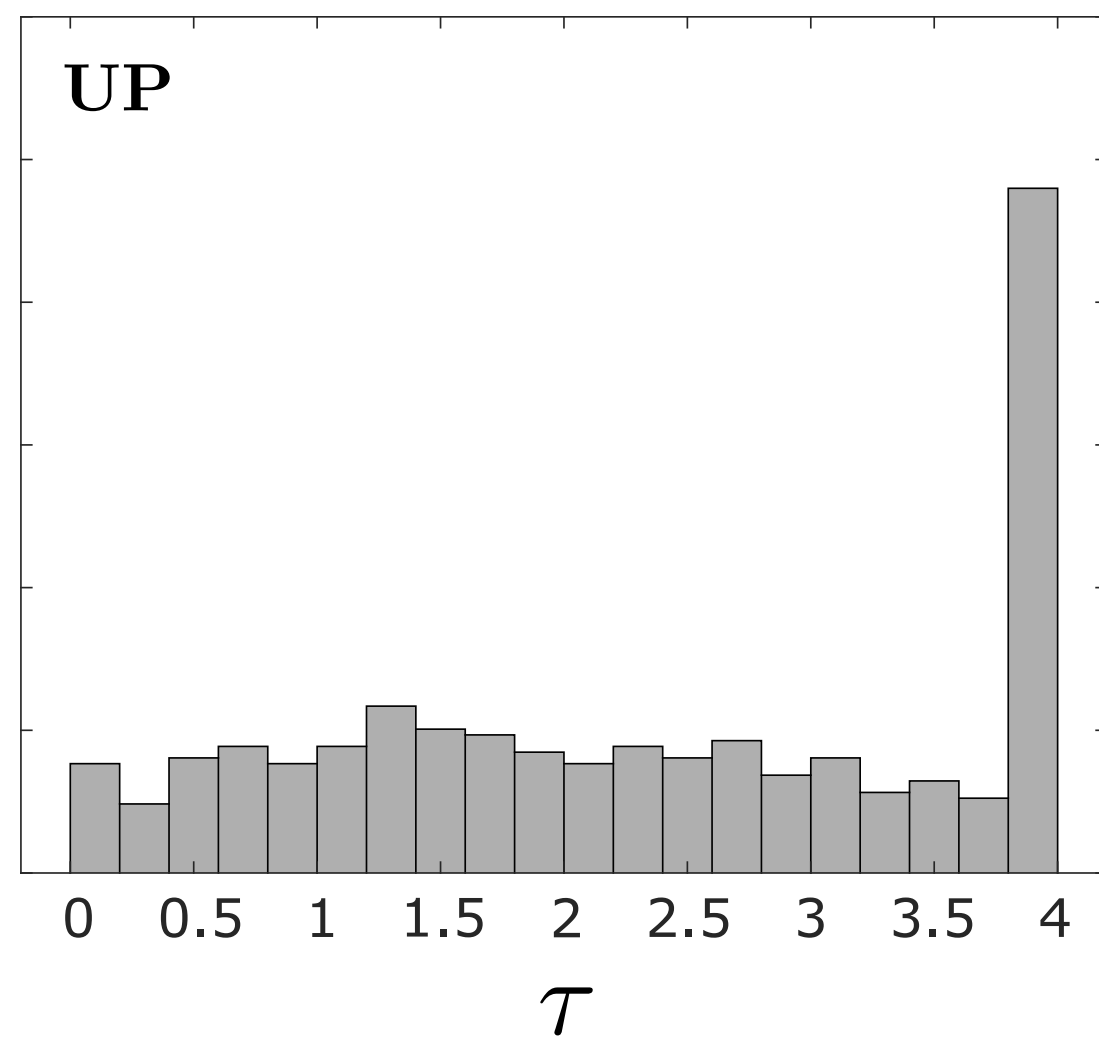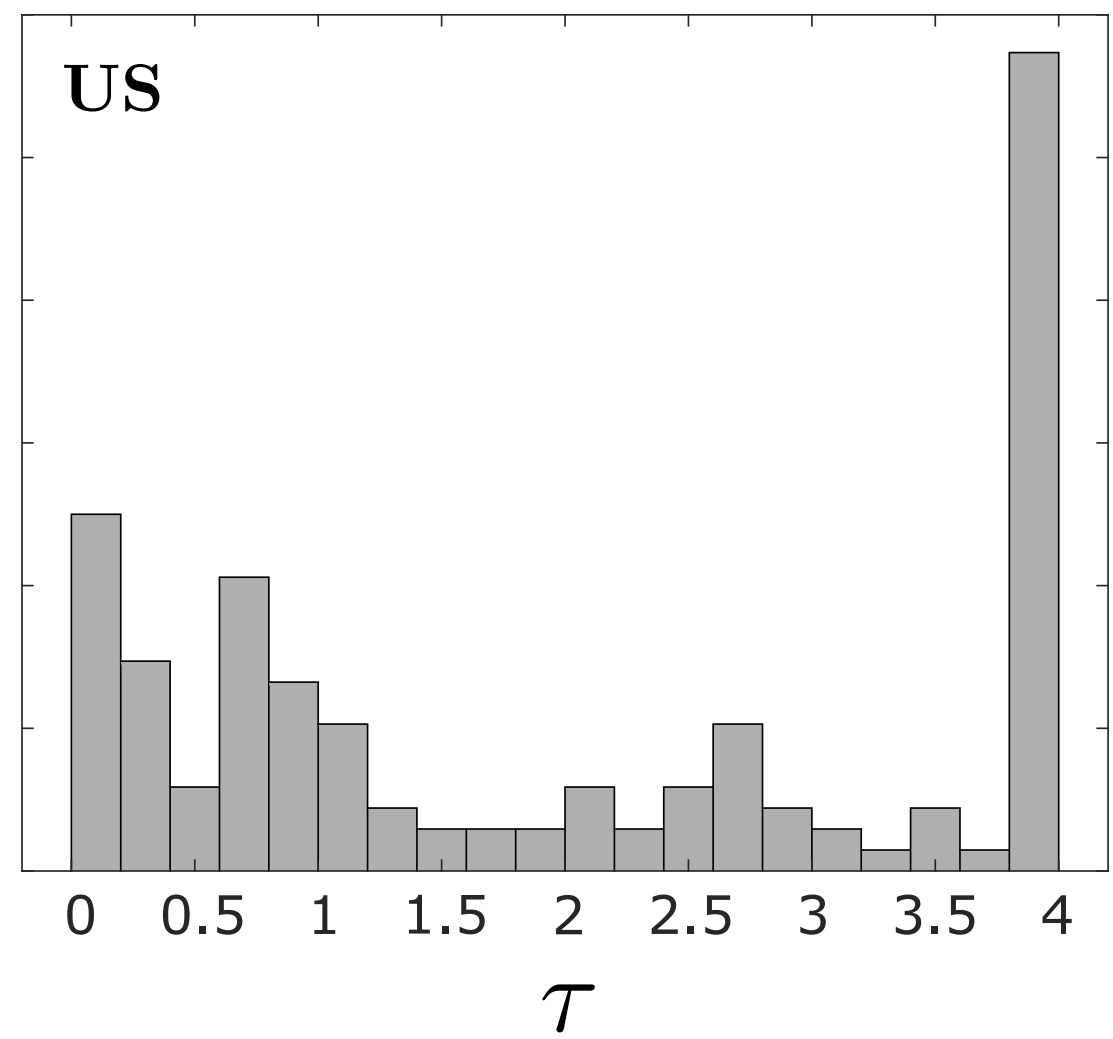

Supplement: S9 Fig — In our model, it is assumed that each single cell starts growing into an organoid on Day −τ, where the parameter τ is allowed to vary between organoids. For each organoid in each dataset, the value of the parameter τ was estimated using the Gompertz model (Sections “Gompertz model” and “Model fitting”), and each panel shows how the estimated values of τ are distributed across the individual organoids for each patient. The distribution of τ is skewed toward τ = 0 for UK organoids and toward τ = 4 for UP organoids. For US organoids, the distribution of τ more resembles a bimodal distribution, where both small and large values of τ are common. (PDF) [file pcbi.1012256.s010.pdf]
