# Supplementary material for: Understanding patient-derived tumor organoid growth through an integrated imaging and mathematical modeling framework
Source: PLoS Comput Biol. 2024 Aug 2;20(8):e1012256. doi: 10.1371/journal.pcbi.1012256 (PMC11324155; doi:10.1371/journal.pcbi.1012256)
Supplement: S1 Table — (PDF) [file pcbi.1012256.s011.pdf]

	UK–1	UK–2	UP–1	UP–2	US–1	US–2	US–3
Number of tracked objects	506	496	1347	641	227	479	535
Objects $\leq 300\ \mu m^2$ in area at any time point removed	330	319	1037	475	130	297	291
Organoids dead at Day 3 or Day 5 removed	281	293	944	420	109	174	145
Only keep organoids growing between each pair of time points	196	226	712	321	75	86	80
Apply merging and splitting data filter described in S1 Text	147	156	306	190	41	50	45
