# Supplementary material for: Understanding patient-derived tumor organoid growth through an integrated imaging and mathematical modeling framework
Source: PLoS Comput Biol. 2024 Aug 2;20(8):e1012256. doi: 10.1371/journal.pcbi.1012256 (PMC11324155; doi:10.1371/journal.pcbi.1012256)
Supplement: S2 Table — The best-fit model for each plate is indicated by bold. (PDF) [file pcbi.1012256.s012.pdf]

	Exp	PL 1/2	PL 2/3	PL 3/4	Gomp	Log	vB 1/2	vB 2/3	vB 3/4
Plate 1	34.02	30.27	28.95	29.16	**20.31**	20.35	26.55	22.90	21.80
Plate 2	27.34	29.52	27.61	26.72	**22.89**	23.84	29.51	27.15	25.86
