# Supplementary material for: Understanding patient-derived tumor organoid growth through an integrated imaging and mathematical modeling framework
Source: PLoS Comput Biol. 2024 Aug 2;20(8):e1012256. doi: 10.1371/journal.pcbi.1012256 (PMC11324155; doi:10.1371/journal.pcbi.1012256)
Supplement: S3 Table — The best-fit model for each plate is indicated by bold. (PDF) [file pcbi.1012256.s013.pdf]

	Gompertz	Logistic	vB 1/2	vB 2/3	vB 3/4
Plate 1	**0.081**	0.086	0.167	0.116	0.102
		1.062	*2.062*	*1.432*	*1.259*
Plate 2	**0.125**	0.143	0.286	0.203	0.172
		1.144	*2.288*	*1.624*	*1.376*
