# Supplementary material for: Understanding patient-derived tumor organoid growth through an integrated imaging and mathematical modeling framework
Source: PLoS Comput Biol. 2024 Aug 2;20(8):e1012256. doi: 10.1371/journal.pcbi.1012256 (PMC11324155; doi:10.1371/journal.pcbi.1012256)
Supplement: Peer review history

# Peer review: Decision Letter 0

**DOI:** 10.1371/journal.pcbi.1012256.r001

**Authors:** Dominik Wodarz, Zhaolei Zhang

11 Jun 2024

Dear Dr. Foo,

We are pleased to inform you that your manuscript 'Understanding patient-derived tumor organoid growth through an integrated imaging and mathematical modeling framework' has been provisionally accepted for publication in PLOS Computational Biology.

The reviewers were very positive about the manuscript. They did make some minor suggestions. Please go through them and modify the manuscript accordingly before uploading the final version.

Before your manuscript can be formally accepted you will need to complete some formatting changes, which you will receive in a follow up email. A member of our team will be in touch with a set of requests.

Please note that your manuscript will not be scheduled for publication until you have made the required changes, so a swift response is appreciated.

IMPORTANT: The editorial review process is now complete. PLOS will only permit corrections to spelling, formatting or significant scientific errors from this point onwards. Requests for major changes, or any which affect the scientific understanding of your work, will cause delays to the publication date of your manuscript.

Should you, your institution's press office or the journal office choose to press release your paper, you will automatically be opted out of early publication. We ask that you notify us now if you or your institution is planning to press release the article. All press must be co-ordinated with PLOS.

Thank you again for supporting Open Access publishing; we are looking forward to publishing your work in PLOS Computational Biology. 

Best regards,

Dominik Wodarz

Academic Editor

PLOS Computational Biology

Zhaolei Zhang

Section Editor

PLOS Computational Biology

***********************************************************

Reviewer's Responses to Questions

**Comments to the Authors:**

**Please note here if the review is uploaded as an attachment.**

Reviewer #1: In this manuscript, Gunnarsson et al provide a comprehensive comparison of multiple existing computational models on patient-derived colorectal tumor organoids in exponential growth.

This paper represents important contribution toward establishing experimental and computational models that can explain organoid growth. As preclinical studies of oncology models move from 2D to 3D cell culture, the work presented herein is likely to be applicable and of interest to a wide audience. Overall, this is a well-written and sound study. I have a few comments and critiques that would strengthen this manuscript.

1. Estimates of mutational burden seem over-interpreted and potentially quite error prone. As the authors indicate, the in vivo/patient setting expects to acquire mutations over years, particularly in the absence of therapeutic stress/selection pressure. The authors should use a more sophisticated model here or else remove these analyses from the paper.

2. The preprocessing of 3D image stacks to support incorporation of quantitative image-based features into a computational model is an important aspect of this study. As such, the authors should provide additional details and code from their image analysis pipelines, i.e. for lines 352-358.

3. Code provided to reproduce findings are included in a publicly accessible GitHub repo; however, also providing a version of the code that a reader could deploy on their own data would further enhance the impact of the paper.

4. The associated raw image data may be of interest to the general community. The authors should comment on how they will make it available.

Reviewer #2: This work evaluates the fitting of data from tumor organoids, obtained by employing a high-throughout image deep learning platform, to various growth models. It is an interesting work, providing with significant insight regarding the tumor growth, while it also reports the inter- and intrapatient heterogeneity in the tumor growth parameters. I suggest the publication of this work after the following minor points are addressed.

1) In the Materials and Methods Section, the three datasets UP, US and UK are introduced. However, these acronyms are not defined, and while the genomic analysis for each sample is reported in Table 1, the main differences (e.g. tumor stage) between the different datasets are not discussed from the beginning. This is important, since some of the results differ between datasets.

2) For the fitting evaluation, the Gompertz model is shown to be the best fit, a fact from which we can infer important conclusions e.g., as the authors mention, "cell division occurring uniformly across the organoid, as opposed to be restricted to the outermost cell layer" (as assumed in the von Bertlanffy model). This is a significant central result of the manuscript. However, in Section 3.3, it is stated that in each dataset, many organoids undergo exponential and not Gomperzian growth. The authors should provide more details on the percentage of organoids undergoing exponential growth, as well as for the carrying capacities of the ones undergoing Gompertzian growth (more detailed than Figure 4b, maybe a distribution of carrying capacities for each dataset?).

3) Building upon the previous point, I am curious to see a discussion on what a carrying capacity in the organoid growth means for the growth of the original tumor itself. Does it mean that the tumor itself will reach a carrying capacity? Isn't it expected for tumors in stage 4 (like the ones in UK and UP datasets) to follow an unbounded growth in volume? Or the carrying capacity for the organoids is translated to a carrying capacity for tumors that is high enough for the tumor growth to be considered exponential? In the final paragraph of Section 3, the authors discuss the difference in timescales between organoid and tumor growth. I think that a similar discussion for the carrying capacities is apposite.

**********

**Have the authors made all data and (if applicable) computational code underlying the findings in their manuscript fully available?**

The PLOS Data policy requires authors to make all data and code underlying the findings described in their manuscript fully available without restriction, with rare exception (please refer to the Data Availability Statement in the manuscript PDF file). The data and code should be provided as part of the manuscript or its supporting information, or deposited to a public repository. For example, in addition to summary statistics, the data points behind means, medians and variance measures should be available. If there are restrictions on publicly sharing data or code —e.g. participant privacy or use of data from a third party—those must be specified.

Reviewer #1: Yes

Reviewer #2: Yes

**********

PLOS authors have the option to publish the peer review history of their article (what does this mean?). If published, this will include your full peer review and any attached files.

If you choose “no”, your identity will remain anonymous but your review may still be made public.

**Do you want your identity to be public for this peer review?** For information about this choice, including consent withdrawal, please see our Privacy Policy.

Reviewer #1: No

Reviewer #2: No

# Editor report: Acceptance letter

**DOI:** 10.1371/journal.pcbi.1012256.r002

**Authors:** Dominik Wodarz, Zhaolei Zhang

26 Jul 2024

PCOMPBIOL-D-24-00726 

Understanding patient-derived tumor organoid growth through an integrated imaging and mathematical modeling framework

Dear Dr Foo,

I am pleased to inform you that your manuscript has been formally accepted for publication in PLOS Computational Biology. Your manuscript is now with our production department and you will be notified of the publication date in due course.

The corresponding author will soon be receiving a typeset proof for review, to ensure errors have not been introduced during production. Please review the PDF proof of your manuscript carefully, as this is the last chance to correct any errors. Please note that major changes, or those which affect the scientific understanding of the work, will likely cause delays to the publication date of your manuscript. 

Soon after your final files are uploaded, unless you have opted out, the early version of your manuscript will be published online. The date of the early version will be your article's publication date. The final article will be published to the same URL, and all versions of the paper will be accessible to readers.

Thank you again for supporting PLOS Computational Biology and open-access publishing. We are looking forward to publishing your work! 

With kind regards,

Lilla Horvath

PLOS Computational Biology | Carlyle House, Carlyle Road, Cambridge CB4 3DN | United Kingdom ploscompbiol@plos.org | Phone +44 (0) 1223-442824 | ploscompbiol.org | @PLOSCompBiol
